# The Convenience of Polydopamine in Designing SERS Biosensors with a Sustainable Prospect for Medical Application

**DOI:** 10.3390/s23104641

**Published:** 2023-05-10

**Authors:** Lulu Tian, Cong Chen, Jing Gong, Qi Han, Yujia Shi, Meiqi Li, Liang Cheng, Lin Wang, Biao Dong

**Affiliations:** 1 Department of Oral Implantology, School and Hospital of Stomatology, Jilin University, Changchun 130021, China; tianll22@mails.jlu.edu.cn (L.T.); gongjing22@mails.jlu.edu.cn (J.G.); hanqi22@mails.jlu.edu.cn (Q.H.);; 2 State Key Laboratory on Integrated Optoelectronics, College of Electronic Science and Engineering, Jilin University, Changchun 130021, China

**Keywords:** polydopamine, surface-enhanced Raman scattering, hybrid materials, biosensors, SERS labels, sustainability

## Abstract

Polydopamine (PDA) is a multifunctional biomimetic material that is friendly to biological organisms and the environment, and surface-enhanced Raman scattering (SERS) sensors have the potential to be reused. Inspired by these two factors, this review summarizes examples of PDA-modified materials at the micron or nanoscale to provide suggestions for designing intelligent and sustainable SERS biosensors that can quickly and accurately monitor disease progression. Undoubtedly, PDA is a kind of double-sided adhesive, introducing various desired metals, Raman signal molecules, recognition components, and diverse sensing platforms to enhance the sensitivity, specificity, repeatability, and practicality of SERS sensors. Particularly, core-shell and chain-like structures could be constructed by PDA facilely, and then combined with microfluidic chips, microarrays, and lateral flow assays to provide excellent references. In addition, PDA membranes with special patterns, and hydrophobic and strong mechanical properties can be used as independent platforms to carry SERS substances. As an organic semiconductor material capable of facilitating charge transfer, PDA may possess the potential for chemical enhancement in SERS. In-depth research on the properties of PDA will be helpful for the development of multi-mode sensing and the integration of diagnosis and treatment.

## 1. Introduction

Engineered nanomaterials need to be designed and synthesized in safe and sustainable ways to unlock their potential global economic, social, and environmental benefits. Therefore, while striving to invent new materials with better properties, an inescapable issue is to mitigate the risks for human health and the environment at all stages of their life cycle, including material extraction and processing, manufacturing, use, and the end of their life. More emphasis should be placed on the following aspects: (1) utilizing ingredients that can be quickly and harmlessly recycled as much as possible; (2) selecting a reaction pathway that consumes less energy and maximizes raw materials utilization; (3) elucidating the detailed mechanism of material synthesis; (4) finely characterizing physicochemical properties of end products; and (5) accurately predicting their short- and long-term biological activity. Among these, using renewable feedstocks is the focus of the beginning of the life cycle and an essential element of the green chemistry principles [1,2].

In the surface-enhanced Raman scattering (SERS) technique, a lot of innovative nanomaterials are invented to enhance the weak Raman scattering intensity of adjacent molecules by electromagnetic enhancement (EE) and chemical enhancement (CE). With the narrow and sharp fingerprint peaks and unique spectral profiles, the information and number of those molecules are clearly presented. Highly sensitive and selective results of qualitative and quantitative detection can be obtained rapidly and minimally invasively, with an acquisition time of only a few seconds and with proper laser power [3,4,5]. Some new SERS sensors achieved the purpose of reuse through cleaning [6,7], heating [8], light decomposition [9], hydrophobic modification [10] of the substrate, and good use of reversible intermolecular force [11]. Recently, it has been popular to design SERS tags, which can stably capture target analytes and output a Raman signal that can be distinguished clearly [12]. Polymers as functional interfaces have excellent structures and properties which can be used to fabricate robust, sensitive, and selective SERS sensors. Smarter sensors are available thanks to the properties of the polymers themselves and the convenience of introducing other functional ingredients in large quantities. For example, being target-matched, stimuli-responsive, non-fouling, electro-conductive, and biocompatible are all key points in designing sensors. Typical structures include (1) hair-like open polymer brushes of repeat units attaching to the core material; (2) surface molecularly imprinted polymer (MIP) coatings with mechanically and thermally robust interconnected structures; (3) layer-by-layer assemblies [13].

Cellulose, starch, silk fibroin, collagen, chitosan, alginate, lignin, and other materials from animals or plants not only have tailorable chemical components and mechanical properties, but also have rich sources, biocompatibility, and biodegradability [14]. They are ideal materials for the preparation of environmentally friendly biosensors. Inspired by the catechol and amino groups of Mytilus edulis foot protein 5 with strong adhesion, polydopamine (PDA) was first discovered as a biomimicry that could coat diverse materials [15]. It is also the main pigment in natural eumelanin, possessing biocompatibility and biodegradability [16]. Typically, the solid substrate is immersed in Tris buffer (pH 8.5) containing a low concentration of dopamine hydrochloride at ambient temperature and pressure for 5–6 h to generate the PDA coating. Compared with other surface modification methods, it requires less and low-cost raw materials, facile operation, and a mild reaction condition. Most importantly, PDA can adhere to almost all organic and inorganic materials, without special surface preparation and aggressive cleaning, and then directly utilize its rich functional groups for secondary modification to obtain the desired properties easily [17].

**Figure 1:**
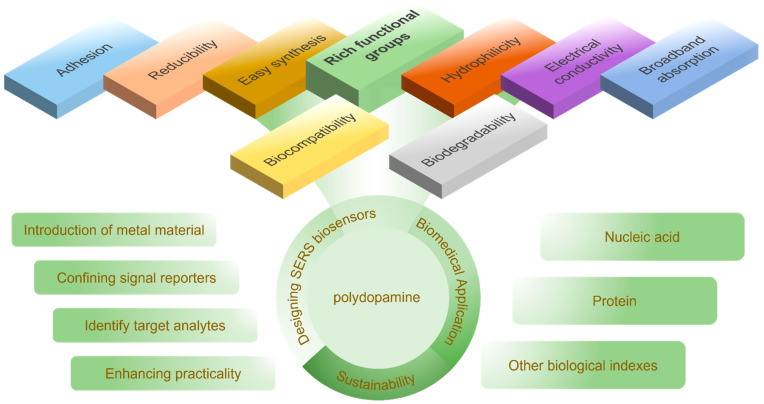
Overview of the various properties of PDA that can be used to design SERS biosensors with sustainable prospects for biomedical applications.

However, considering the simplified synthesis step and multiple performance for practical and sustainable utilization, this review focused on the advantages of PDA as a polymer in designing SERS sensors and its application in the biomedical field (Figure 1) in recent years. Firstly, the mechanism of SERS, and the molecular structure and synthesis techniques of PDA will be briefly described. Secondly, guidance for the design of SERS sensors will be proposed: (1) the introduction of noble metal or semiconductor materials; (2) the strategies for loading abundant signal molecules; (3) the methods for facilitating the identification of target objects; (4) the routes for enhancing the practicality of the biosensor. Thirdly, recent cases of SERS biosensors in diagnosis will be summarized. Finally, it is hoped that this review will inspire us to spare no efforts to explore more properties and a more detailed theory of PDA to enhance the intensity of Raman scattering, and to stimulate its potential in combination with other sensing and therapeutic modalities through flexible conception and simple synthesis.

## 2. Mechanism of SERS

The Raman scattering effect was discovered by Indian scientist C. V. Raman in 1928 [18]. Under the excitation of light, there is little scattered light, mainly changing the vibrational energy. In the scattering process, the molecule absorbs energy and jumps from the initial state to a virtual energy level, and then falls back to a lower energy level and emits scattered photons. This phenomenon is also called inelastic scattering and includes Stokes and anti-Stokes scattering. The Stokes scattering leads to a smaller frequency of the emitted photons than that of the excitation source and red-shifted inelastic scattering. Anti-Stokes Raman scattering is the opposite [19]. The shift of inelastic scattered light with respect to the applied excitation wavelength appears together on the Raman spectrum. The fingerprint-like Raman spectrum reflects the intrinsic molecular vibrational information for determining the chemical components, the molecular conformation, and the interaction between molecules. Additionally, commendably, the interference signal from water in the sample can be negligible under the excitation of the visible and near-infrared light [5]. Unfortunately, only 10^−8^ of the incident photons are scattered inelastically [19], and the Raman scattering cross-section is merely about 10^−30^–10^−25^ cm^2^ per molecule [20], resulting in poor sensitivity.

In 1974, Fleischmann et al. found that a Ag electrode with a rough surface enhanced the Raman signal of pyridine adsorbed on it [21]. In 1977, Van Duyne et al. theoretically calculated that the Raman signal of each pyridine molecule was enhanced by 10^5^–10^6^ and initially called this phenomenon surface Raman scattering [22]. Considering the low and indistinguishable Raman scattering, noble metals (e.g., Au/Ag/Cu) and semiconductor materials (e.g., graphene and its analogues, transition metal sulfides or oxides, conjugated organic compounds) [23] are used to adsorb target molecules on their surfaces, resulting in bright Raman signals via EE or CE. Compared to the fluorescence (FL) signal, SERS has higher sensitivity (even down to the single molecule level) and better selectivity (obtaining multiple peaks with narrow bandwidth for multiplex detection) under single laser irradiation. Besides, SERS sensors are more suitable for long-term utilization due to the ability of SERS signal molecules to resist photobleaching and photodegradation [5]. Meaningfully, damage to SERS-active substances and molecules from the laser should be paid more attention to [24,25]. It is necessary to reduce the power of the laser, shorten the irradiation time, and use the laser with longer wavelengths. The metal–semiconductor complexes can be affected by these parameters minimally, according to some examples of reusable SERS sensors. A label-free SERS tag, Fe_3_O_4_@TiO_2_@Ag, could be reused for nine cycles to detect prostate specific antigen under the laser with a power of 20 mW and an integration time of 10 s. The UV lamp (365 nm, 6 W cm^−2^) used for the photocatalytic degradation of targets caused little damage to it [9]. Moreover, Chi et al. observed that monolayer graphene could retain the original shape of Au triangular nanoarrays for 16 annealing cycles [26]. However, the nanoporous structures were worn out after 22 reuse cycles, shown by the SEM photo [27]. Actually, the SERS activity of most materials decreases significantly after a few cycles [7,8]. The source of this destruction can be further investigated and solved for the development of sustainable SERS sensors. Apart from that, the protection of molecules from photo-induced invasion is crucial for medical application [28]. SERS substrates can be optimized for the excitation of near-infrared light to reduce the light damage to biological tissues [29] and export the detection result of less autofluorescence interference [30].

**Figure 2:**
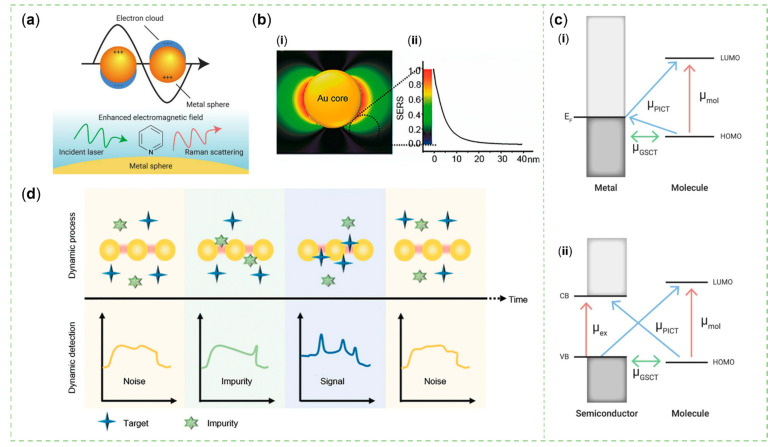
Mechanism of SERS about EE and CE. (**a**) Electromagnetic enhancement in SERS based on plasmonic nanospheres [23]. (**b**) Dependence of the electric field (**i**) and SERS (**ii**) enhancement on the distance from Au surfaces [5]. (**c**) Charge transfer transitions in metal–molecule (**i**) and semiconductor–molecule systems (**ii**). (Some notes for the abbreviations in the picture. E_F_: the Fermi level in the metal; µ_GSCT_: the interfacial ground-state charge transfer; µ_PICT_: the photoinduced charge transfer resonance; µ_ex_: exciton resonance in the semiconductor; µ_mol_: molecule resonance.) [23] (**d**) A Challenge from the dynamic process of targets and impurities moving into the hot spot for qualitative and quantitative analysis of SERS substances [5]. Copyrights: all images have been adapted and reproduced with permission from: (**a**,**c**) © 2020 Shan Cong et al. published by Elsevier; (**b**,**d**) © 2018, American Chemical Society.

The SERS effect is regarded as the result of a combination of the enhanced electric field around the molecule and the increased molecular polarizability, and can be explained by two classical theoretical models, EE and CE [31]. In the theory of EE (Figure 2a,b), this electric field is usually composed of two steps. Firstly, the nanoparticle (NP) can be excited by a field at the incident wavelength to create a local field. Secondly, the molecule at the vicinity of the NPs can be polarized by the local field to produce a scattered field at the Raman wavelength. Then, the scattered field can interact with the NP to create a re-radiated field [5,32]. The intensity of the surface electric field is negatively correlated with the distance of molecules from the metal surface [33,34]. The enhancement of the local electric field mainly comes from the localized surface plasmon resonance (LSPR) of the noble metal. When the frequency of the incident light matches the natural oscillation frequency of the free electrons in the metal, the electromagnetic wave can drive the electrons of the conduction band (CB) at the metal–dielectric interface to produce collective oscillation, and LSPR will occur when it is highly localized to a specific site [4,5,35]. Moreover, when the diameter of semiconductor NPs without significant LSPR is close to the wavelength of the incident laser, Mie scattering theory cannot be ignored. This phenomenon is related to the resonance of charges with a three-dimensional distribution within the NP [36,37]. Mie resonances depend on the refractive index contrast between the dielectric sphere and the surrounding dielectric to affect the spatial distribution of the electromagnetic field. Particles with cavity structures or larger particle sizes will exhibit better performance in this resonance [38,39,40,41]. The CE is relatively weak compared to the EE, generally induced by the formation of chemical bonds, the resonance, and photo-induced charge transfer (PICT) between the adsorbed molecule and the substrate [42,43,44]. Notably, PICT (Figure 2c) is related to the charge transfer between the lowest unoccupied molecular orbital (LUMO) or the highest unoccupied molecular orbital (HUMO) of the molecule, and the Fermi level of a metal or the CB and valence band (VB) of a semiconductor [3,23]. Moreover, the coupled resonance strategy should be researched while designing novel SERS substances containing semiconductors. In this theory, it is suggested that two or more of the three factors—molecule-semiconductor CT resonance, molecular or exciton resonance, and plasmon or Mie scattering resonance—are at or near the laser excitation wavelength simultaneously.

The activity of the SERS substrate was related to its size, thickness, shape, structure (such as gap, tip, and edge) and refraction coefficient. Regrettably, the irregular morphology of NPs is incompatible with their uniform dispersion, both of which have a positive effect on SERS activity. Meanwhile, colloidal plasmonic metal NPs tend to aggregate and have a short service life [3], which makes the repeatability of the SERS spectrum poor while the quantitative values of multiple sites must be recorded in a specific time window to be calculated to obtain an average. The formation of dimers contributes to a high electric field intensity, namely one kind of hot spot, due to the gap of 2–10 nm [4]. If the sample content is too small [5] and the gap between two particles is too small [4], the unreliability of the detection structure will be increased (Figure 2d). Therefore, designing a SERS substrate with a more elaborate structure is considered to be a reliable means to improve the sensitivity, specificity, and reproducibility of SERS. The Raman signal of the final detection result can come from the target detection molecule itself or from the signal molecule pre-incorporated in SERS tags that have captured the target molecule. To form relatively complete SERS labels, the following components should be included: metal or semiconductor materials, Raman reporter molecules, protective shells, and identification elements [12].

## 3. Synthesis and Molecular Structure of PDA

PDA stands out among the many polymers for surface functionalization because of its easy polymerization and many other excellent properties. Electropolymerization, enzymatic oxidation, and solution oxidation are simple methods for preparing PDA without the need for sophisticated equipment. We can select different buffers and solvents, regulate pH value and temperature, add various oxidants, change dopamine (DA) concentration and supporting substrates, and introduce external stimuli to obtain PDA film with ideal thickness and roughness [45].

**Figure 3:**
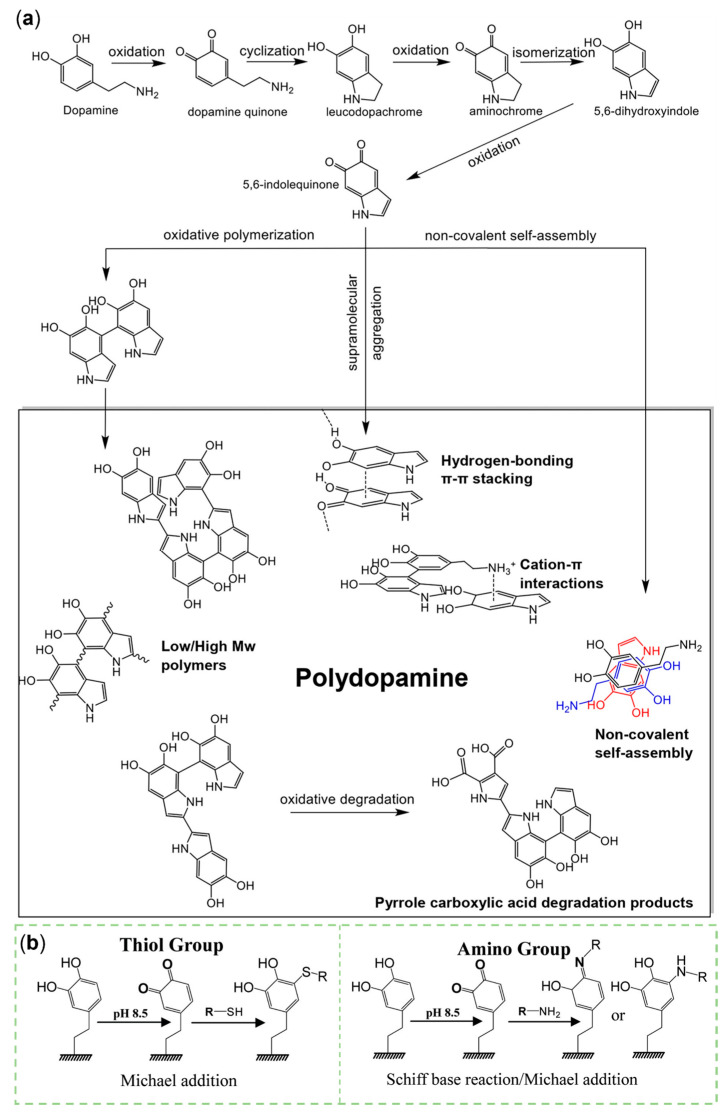
(**a**) Diagram of the synthesis mechanism of PDA [47]. (**b**) Schematic illustration of the Michael addition or Schiff base reaction [53]. Copyrights: all images have been adapted and reproduced with permission from: (**a**) © 2022 Elsevier B.V; (**b**) © 2010, American Chemical Society.

The detailed molecular structure of PDA is not clear from current research. It is generally believed that PDA could be obtained by covalent polymerization and non-covalent self-assembly [46]. In the process of DA oxidation polymerization, various small-molecule intermediates are formed, e.g., DA, dopamine quinone, leucodopachrome, aminochrome, 5,6-dihydroxyindole (DHI), and 5,6-indolequinone (Figure 3a) [47]. Including these small molecules, the final products may also consist of oligomeric components and high-molecular-weight polymers [45]. By C-C bonding, hydrogen bonding, π-π stacking, and cation–π interactions, each monomer is linked [48]. Trimers of the (DA)_2_/DHI complex, via non-covalent stacking [46,49] and flake and cyclic tetramers formed by the C-C bond, are two distinct oligomers [50,51]. Under basic conditions with hydrogen peroxide, the catechol or o-quinone ring can be transformed to pyrrole carboxylic acids by oxidative degradation. Each part is the breakthrough point for probing the properties of PDA, including amino, imine, catechol, quinone, carboxy functional groups, and benzene rings. PDA is a versatile material that can be used to design SERS sensors. Adhesion is one of its most prominent and well-studied properties. With diverse functional moieties, it can be coated on precious metals, semiconductors, oxides, polymers, and ceramics with complex morphology through metal coordination or chelation, hydrogen bonding, π-π stacking, and cation–π interactions [15,16,17,52]. The most commonly used form of covalent adhesion is the introduction of sulfhydryl by Michael addition and of amino by Michael addition or Schiff base reaction (Figure 3b) [53]. This kind of chemical reaction and the reducibility of PDA are attributed to the quinone moiety. As a zwitterion, PDA can possess a positive charge at a lower pH for the protonated amino groups and a negative charge at a higher pH for the deprotonated catechol groups. The electrical conductivity and paramagnetic properties of PDA can be regulated by the π-electron. PDA is usually hydrophilic with low surface energy and can absorb the energy of light ranging from UV to infrared to protect molecules from damage [16,48]. Due to the satisfying biocompatibility and biodegradability, PDA is a burgeoning material in the biomedical field [16].

The molecular structure, thickness, and roughness of PDA depend strongly on the mode of synthesis [48]. The following cases can serve as references for synthesizing of PDA-modified nanomaterials to improve the SERS properties. The thickness of PDA is positively correlated with the concentration of DA and the pH value of the solution. The use of a low concentration of DA and multiple coating steps by shortening each immersion time contributes to inhibiting the formation and aggregation of PDA particles to reduce the roughness of the PDA film [17]. For example, Badillo-Ramirez et al. used SERS characterization technology to observe the changes in peak value on different bands. They also proved that when PDA was covered with Ag colloidal particles reduced by varied reducing agents, laser wavelength, irradiation time, and pH would produce diverse molecular structures in PDA [54]. Alfieri et al. found that the pre-existing PDA membrane was able to adsorb DA and other intermediate monomers and oligomers in solution, even at low concentrations. And in the polymerization system with periodate oxidizer, the conversion rate of catechol to quinone groups was found to be higher compared to that in the autoxidation system. It was also suggested that oxidation fission into carboxyl groups may occur. Long chain aliphatic amines provided hydrophobic moieties, inhibited intramolecular cyclization and the formation of large insoluble aggregates to promote the growth of PDA films, and thus improved underwater adhesion [55]. Intriguingly, biomolecules such as proteins, peptide chains, and nucleotides can also control DA oxidation and aggregation and enhance the stability and biocompatibility of PDA. The products of oxidative polymerization are diverse due to the interaction between oligomeric species and the bases of DNA, electrostatic attraction, and spatial complementarity [47]. Moreover, Raman spectra reflect the existence of PDA, especially the two obvious wideband peaks, near 1365 cm^−1^ and 1575 cm^−1^ [56], covering the signals of target molecules [57]. The Raman peaks from the undesired materials are difficult to circumvent. Amine-free organic (e.g., bicine) or inorganic (e.g., phosphate) buffers can be used as alternatives to avoid mixing Tris into the end product. Microwave irradiation can speed up the reaction rate [17].

According to the requirements of green synthesis, the amount and type of ingredients fed into the reaction system need to be minimized and simplified not to affect the purity of the final product, and must also be environmentally friendly. Given the prospect of mass production, the utilization of external power for the reaction system can improve the reaction efficiency. However, unreasonable energy supply goes against the concept of sustainable development. Notably, an increasing number of researchers are anticipated to concentrate on investigating the molecular structure and functionality of PDA. At present, PDA mainly played an auxiliary role in designing SERS substrates and failed to play a direct role in Raman signal enhancement, although PDA as an organic semiconductor material has such potential.

## 4. Guideline for Designing SERS Substrates Using PDA

Nanomaterials for SERS biosensing can be divided into three types in modality. Firstly, the label-free strategy can directly bond biological components to the material surface, but it is very difficult to select the characteristic peaks of target analytes for qualitative and quantitative analysis. Secondly, in the reaction-based strategy, with the occurrence and variation of the surrounding chemical composition, the functional groups of the probe molecules attached to the interface of the nanostructure will change, reflected in the Raman spectrum. Thirdly, the fabrication of SERS tags is currently a prevalent practice in the field [58]. The most important point is that the Raman characteristic peaks enhanced by SERS substrates for identifying and combining analytes can come from the analytes intrinsically, or from the reporters extrinsically in SERS labels [3]. Enlightened by the review of Gong et al. [12], this paper will firstly discuss the convenience brought by PDA in designing SERS tags containing metal or semiconductor materials, Raman reporters, and recognition elements. Then, the advantages of PDA will be revealed from the standpoint of enhancing the practicality of the SERS sensor.

### 4.1. Introduction of Metal or Semiconductor Materials

At present, metal and semiconductor materials are an essential part of each SERS substrate. PDA can self-polymerize on pre-synthesized materials to form coatings of appropriate thickness and rough morphology. Amino and catechol groups play an important role in fixing and reducing metal ions to form crystal seeds without other reducing agents, and a uniformly distributed metal matrix is eventually obtained. Just like glue, PDA has great convenience and charm in synthesizing a variety of metal or semiconductor materials into beaded, core-shell, or other peculiar configurations that help to heighten SERS activity. In addition, noble metals are expensive and in short supply, so the exploration of alternative materials in the SERS field is highly anticipated. For example, some semiconductor substrates with lower enhancement factors than noble metals can be improved by oxygen incorporation and extraction [59]. In this case, the reducibility of PDA can strengthen the stability of the material. It has also been found that PDA can assist in charge transfer and redistribution. There are a few relevant pieces of literature on this aspect, which are worthy of further study.

#### 4.1.1. Adhesive Property

Adhesion is one of the most special properties of PDA. PDA can be a main material, providing ample surface area for other components to adhere to. Alternatively, it can be an auxiliary material by adhering to the core component for complex configurations. In virtue of abundant catechol and amino groups on the PDA nanosphere, several small Au seeds could be deposited for subsequent growth of a Au shell layer with a rough surface topography and numerous voids [60]. Additionally, PDA could adhere to linear [61], spherical [62], cuboid [63], rod-shaped [64], and other forms of materials with tips and sharp edges.

**Figure 4:**
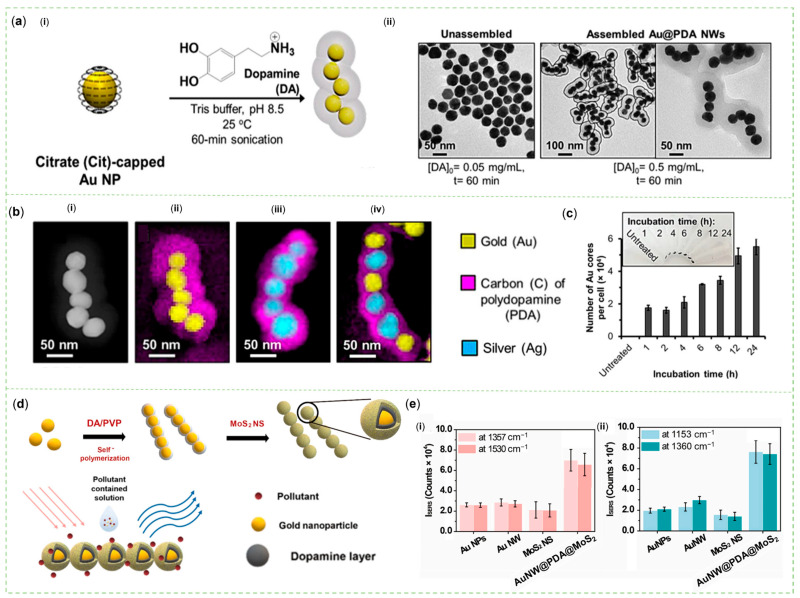
Worm-like SERS tags constructed by PDA. (**a**) (**i**) Conventional diagram of DA-mediated assembly of 40 nm citrate-capped Au (Cit-Au NPs) into Au@PDA nanoworms (NWs). (**ii**) TEM images of unassembled Au NPs and assembled Au@PDA NWs under different concentrations of DA. Black lines outline the morphology of individual NW. (**b**) (**i**) HAADF-STEM image of an Au@PDA NW and (**ii**) EDX elemental map of an Au@PDA NW, (**iii**) an Ag@PDA NW, and (**iv**) a bimetallic Au/Ag@PDA NW. (**c**) Kinetics of the association of Au@PDA NWs to HeLa cells through collecting cell pellets for ICP-MS after treatment with different incubation times [65]. (**d**) Schematic representation of the synthesis of Au NWs@PDA@Molybdenum disulfide (MoS_2_) nanohybrid material and its application for SERS biosensing. (**e**) SERS intensity of two critical Raman peaks with (**i**) Rhodamine B (RhB) at 1357 and 1530 cm^−1^ and (**ii**) Methylene blue (MB) at 1153, 1360 cm^−1^ [66]. Copyrights: all images have been adapted and reproduced with permission from: (**a**–**c**) © 2019, American Chemical Society, (**d**,**e**) © 2022 Elsevier B.V.

A moderate amount of PDA could connect 4–5 plasmonic nanospheres into a stable worm-like structure with a large surface area and adjustable LSPR responses. Choi et al. demonstrated that protonated DA of a certain concentration bound to Cit-Au NPs (Figure 4a) or Ag NPs (Figure 4b) in a mildly alkaline environment and subsequently polymerized to form Au/Ag@PDA NWs by facile sonication. Hydrogen bonds between N atoms of DA [shorted as N(DA)] and O atoms of Cit [O(Cit)], O(DA), and O(Cit), as well as N(DA) and N(DA) were essential for this assembly, as proven by the molecular dynamics simulation of intermolecular interactions. In addition, the electrostatic attraction between protonated primary amine groups of DA and carboxyl groups of Cit anions was strong. In their experiments, well-dispersed PDA-coated Au NPs could be synthesized by capping Au cores with anionic ligands, such as tannic acid, carboxylate-terminated or methoxy-terminated poly (ethylene glycol) (PEG). However, compared with Cit-Au NWs, these nanomaterials did not have a special worm-like structure and a second pronounced plasmonic peak. Beyond that, coating Cit-Au cores with some structural analogues of DA, e.g., norepinephrine, epinephrine, catechol, and 3,4-dimethoxyphenethylamine, without the primary amine or catechol group, even led to products without complete shells. As a result, Cit as a capping ligand and DA with primary amine and catechol groups were necessary compositions to form such a characteristic nanohybrid. More importantly, as opposed to granular and rod-like structures, more one-dimensional nanochains protected by PDA with good biocompatibility could enter cells (Figure 4c) via micropinocytosis and not be damaged for up to 24 h. They also scattered red light for label-free, dark-field scattering cell imaging, carried water-insoluble fluorescent dyes for FL imaging, and absorbed near-infrared light for photothermal therapy [65]. What makes their research worth learning from is that they explored the detailed intermolecular forces that ensured the stability of a particular structure and demonstrated that the selected materials were not substitutable. The worm-like nanomaterials had the advantage of entering cells for detection, and had the potential for SERS applications. Later, inspired by this configuration, some researchers adjusted the thickness of PDA and added 2D MoS_2_ nanosheets (NSs) to construct Au NWs@PDA@MoS_2_ nanohybrids (Figure 4d) for SERS sensing. RhB and MB were used to detect its SERS activity, which showed that this combination had a better effect than either component used alone (Figure 4e) [66].

In addition to the unique structure of the chain, PDA also shows great involvement in the construction of petal-like, porous, hollow nanomaterials with large surface areas. Park et al. incubated CuSO_4_ and dopamine hydrochloride in PBS for 3 days at room temperature. Cu^2+^ catalyzed the polymerization of DA to PDA. At the same time, Cu^2+^ bound to the free amino group of PDA, which made Cu_3_(PO_4_)_2_ seeds adhere to the surface of PDA for further anisotropic growth. A large number of Ag^+^ were reduced by the catechol part of PDA to attach to Cu_3_(PO_4_)_2_ nanoflowers (NFs) formed in the previous step. The SERS ship prepared by the NFs coated on a silicon wafer could be reused three times to detect the content of thiocholine, the hydrolyzed product of acetylcholine, in the extracellular fluid of patients with pesticide poisoning, and its limit of detection (LOD) value was 60.0 pM [67]. Mo_7_O_24_^6−^ anions were chelated with catechol groups to form hydrophobic units, while amino groups formed hydrophilic units. After the addition of ammonia hydroxide, these Mo–DA with nanobubbles, dispersed in an ethanol solution, could polymerize into a porous Mo–PDA complex after the addition of ammonia hydroxide. After that, Fe ions could also be chelated to PDA and Au NPs could be captured by amino groups [68]. PDA was attached to the template material of different sizes, shapes, and compositions, and the template was etched off by a hydrothermal method to obtain a spherical, elliptical hollow or yolk-shell structure. During the above process, Wang et al. used the SiO_2_ nanosphere loaded with Au NPs as the template. After etching, Au NPs would remain on the inner wall of the hollow PDA nanospheres, and the outer wall for secondary modification could continue to load plasmonic metals, which was conducive to achieving stronger SERS activity. It was also a way to introduce other components for drug delivery, catalysis, bioimaging, and other functions [69].

The materials with fancy configurations for highly active SERS and strong adaptability for biological applications still based on PDA can be adhered to. Their species need to be expanded and their mechanisms of adhesion should be explored. PDA coated on the Ti_3_C_2_ surface with hydroxyl groups after etching could further absorb Cd^2+^ by its catechol and amine groups [70]. Mn:ZnCdS quantum dots relying on carboxylation beforehand were connected to the amino group in the PDA [71]. The hydrophilic PDA layer could improve the dispersion of Fe_3_O_4_ NPs in solution [72]. PDA could self-polymerize on natural and cheap reduced graphene oxide including the many functional groups of epoxy, carbonyl, and carboxylic acid that could replace expensive noble metals in order to devise novel SERS substrates [73]. Metal organic frameworks (MOFs) with a porous structure and special functional groups are considered as a variety of ideal capture for analytes, and have a remarkable application prospect in the SERS field. The rich amino and phenolic hydroxyl groups in PDA could trap the metal ions of MOFs [72]. UIO-66(NH_2_) was built by Zr^4+^ and 2-aminoterephthalic acid, which could be wrapped by PDA with excellent adhesiveness. Ag NPs and MIP could be modified on PDA. The PDA layer needed to be thin to maintain the original octahedral form of the MOFs, not to reduce “hot spots” and ensure a conspicuous SERS signal [74]. Special phenolic hydroxyl groups of PDA could capture Zn^2+^ for clusters of ZnO nanorods (NRs) [75].

As mentioned above, noble metals, semiconductors, quantum dots, MOFs, and other components can be introduced with the help of PDA to reveal their SERS capability. SERS substrates often require a unique structural design. On the one hand, PDA with a hollow or porous structure as the main supporting component has a large surface area for late modification. On the other hand, PDA as an auxiliary material can be attached to different forms of the main material for remodification. Most notably, the PDA-coated NPs also form stable chains that allow cells to swallow them. When we design the complex SERS substrates and study the Raman signal enhancement ability of new materials, PDA is like a nano-level double-sided adhesive with simple synthesis and varied performance.

#### 4.1.2. Reductive Property

The brilliant reductive property of PDA for metallic cations has been attributed to the electrons released during the oxidation of its catechol groups to the quinone groups [16]. The strong reducibility enabled PDA to introduce Au [76], Ag [61,77,78], and other common noble metals [79] into SERS substrates. PDA nanospheres have the ability to provide a large number of attachment sites for metal particles. Ag particles with a diameter of 10 nm were reduced and then distributed evenly on a PDA nanosphere of about 400 nm. The gap between each Ag particle was close to its radius. Compared with the Ag particles of the same size freely distributed, this design obtained a greater electromagnetic field intensity, which was conducive to SERS signal amplification. Furthermore, in the results from X-ray photoelectron spectroscopy (XPS), after the material was stored at 4 °C for 45 days, the characteristic peaks of Ag ions were not obviously different from those peaks observed in the freshly prepared ones. This demonstrated that PDA could help to maintain the stability of this SERS sensor for long-time utilization [80]. Wang et al. proved that PDA coated on the hydrophobic plastic well plates could also offer electrons to Ag ions. Then, small Ag seeds synthesized in advance as surface-bound catalysts were imperative for the electroless deposition of Ag with uniform size distribution [81]. In addition to the enhanced SERS performance, non-aggregated Ag NPs showed a stronger FL quenching ability to realize the dual-mode sensing combined by SERS and FL [82].

**Figure 5:**
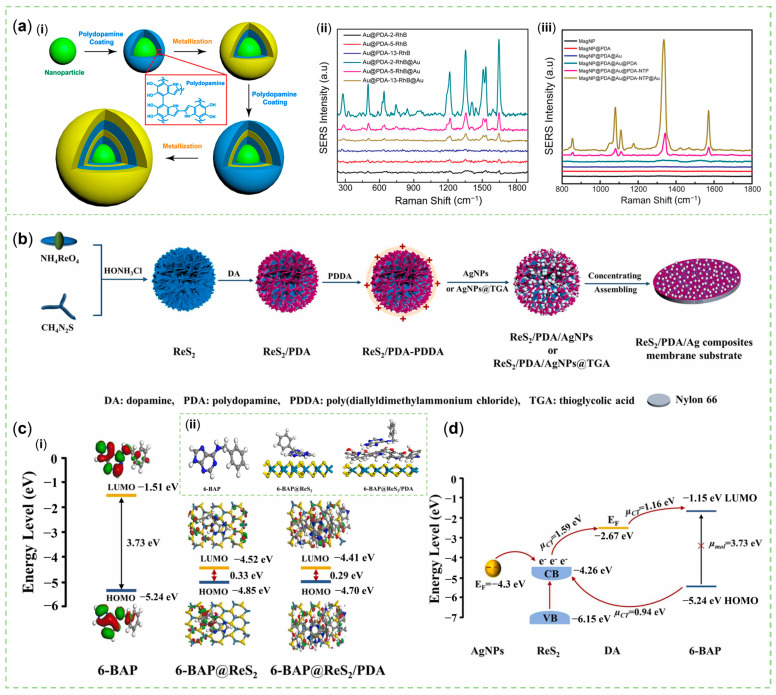
The function of PDA in building a nice structure with nano gaps and a case accounting for the process of charge transfer by PDA. (**a**) (**i**) Schematic illustration of the synthesis of versatile plasmonic nanogapped NPs based on PDA coating. (**ii**) SERS spectra of different Au nanostructures with RhB tags positioned on the PDA layer. (**iii**) SERS spectra of 4-nitrothiophenol (NTP)-encoded magnetic nanogapped NPs and the control NPs [85]. (**b**) A synthetic flow diagram of rhenium disulfide (ReS_2_)/PDA/Ag. (**c**) (**i**) Effect of ReS_2_/PDA for the band gap of 6-benzylaminopurine (6-BAP). (**ii**) The interaction models between ReS_2_/PDA and 6-BAP of density functional theory calculation for HOMO and LUMO. (**d**) The route map of charge transfer in the composites of ReS_2_/PDA/Ag and 6-BAP [86]. Copyrights: all images have been adapted and reproduced with permission from: (**a**) © 2019, American Chemical Society; (**b**–**d**) © 2023 Elsevier B.V.

Notably, core-shell SERS tags were popular because the gap-enhanced Raman effect could be stably obtained and the signal molecules could be placed between gaps without being affected by the harsh and changeable environment of the detected samples [83]. PDA, like double-sided tape, could easily introduce several metal shells and adjust the gap thickness [84]. Intriguingly, PDA could use its reductive property to help add plasmonic materials through layer by layer to form multi-shell NPs with nanogaps of controllable size such as a rocking snowball for ultrahigh SERS activity (Figure 5a) [85].

Uniquely, Jiao et al., designed a yolk-shell Fe/Fe_4_N@Pd/C magnetic nanocomposite as a recyclable SERS sensor. Through annealing at a high temperature under a H_2_/Ar atmosphere, the PDA layer with Pd^2+^ coated on the Fe_3_O_4_ nanospheres with citrate groups became a porous N-doped defective carbon shell. Owing to the reductive property of PDA and H_2_, Pd NPs were synthesized successfully and dispersed uniformly in the carbon shell. Meanwhile, it has good electrical conductivity for electrocatalytic application due to interfacial charge polarization formed between the defective carbon shell and Pd NPs. The carbon layer accumulated electrons while Pd NPs accumulated holes. Eventually, the following three factors, positively charged Pd NPs attracting more rhodamine 6G (R6G) molecules, the electromagnetic field between individual Pd NPs, and the magnetic field from iron cores contributed to the excellent SERS performance synergistically. Moreover, the PDA shell could effectively shield the iron cores from oxidation and maintain material stability, even if its performance was slightly compromised due to damage to its integrity in this work [79].

More oxygen vacancies for metal oxides had been proved to be able to help semiconductor materials to obtain a stronger SERS effect [87]. However, the reaction conditions were rather difficult to obtain, such as calcination in a hydrogen atmosphere, calcination in argon by grinding and mixing with NaBH_4_, and pulse laser irradiation [88]. Worse still, the painstakingly synthesized materials were easily oxidized, causing them to lose their valuable properties. PDA was found to be a green material that could facilely introduce and protect oxygen vacancies to synthesize MoO_3−x_ with a tunable color and LSPR. In a certain range, the higher the pH value in the environment, the more reducing DA became. However, it was still necessary to consider and find semiconductor materials with an appropriate oxygen vacancy for the suitable CB and VB, and proper signal molecules with the LUMO and HOMO. One of the two possible PICT transitions, from the VB to the LUMO or from the CB to the HOMO, should match the excitation light energy to realize charge transfer and molecular resonance, so as to yield the most striking SERS signal [89].

The reducing and antioxidant properties of PDA have been demonstrated in the process of synthesis and protection of noble metals and metal oxides, even in the case where PDA was calcined to form a nitrogen-containing carbon layer. Noble metals are still the ideal materials to achieve strong SERS activity. The reduction of metal ions on the PDA layer is a convenient way to obtain a stable metal layer. Additionally, in the search for alternatives to expensive precious metals, PDA can obviously solve the problem of the easy oxidation of materials.

#### 4.1.3. Charge Transfer

CE is a short-range effect, so there are two critical conditions for the acquisition of efficient charge transfer. One is that the chemical bonding needs to be formed between molecules and the substance for the flow of charge. The other is that the energy level of the substrate needs to match that of the molecule. Importantly, the energy of the incident light should be greater than the minimum required for charge transfer [23]. While conducting a popular multicomponent SERS substance with the couple resonance effect, the inclusion of materials with mismatched energy levels may inhibit this transfer [90,91]. In addition, research on tunning morphology [87], doping elements [92], and creating crystal defects [93] in metal and semiconductor components has proliferated. However, the development of SERS platforms based on π-conjugated organic semiconductors is still in its infancy. A PDA coating that can not only tightly wrap the semiconductor material part but also promote the proximity between the substrate and the analyte is a prerequisite [86]. Many past experiments have proven that for overcoming the shortcomings of wide band-gap semiconductor materials that do not respond to visible and near-infrared light, PDA with strong light harvesting and broadband-absorption ability can be a charge transfer mediator [94,95,96,97]. Selecting this kind of nano-glue that can transfer electrons through a conjugated π structure [94], catechol, or quinone groups [98] may be a shortcut.

As previously mentioned, the utilization of PDA in the research of Yuan et al. played a pivotal role in constructing a distinctive and logical plasmon-coupled 2D nanohybrid system consisting of Au NPs and MoS_2_ NSs. On the interfaces of AuNPs prepared by citrate reduction, the polymerization of DA was controlled by polyvinyl pyrrolidone (PVP) to reduce the PDA thickness. The TEM images showed that worm-like structures were formed with a PDA thickness of 2 nm and an interspace within 1 nm, as well as a high dispersion due to the steric hindrance effect of PVP. Using finite-difference time-domain calculations, multiple hot spots could be observed between each NP. The SERS effect could be enhanced by charge transfer after further loading of MoS_2_ NSs on the Au NWs. The ultrathin PDA between Au NWs and MoS_2_ NSs is an electroactive molecule [66]. Additionally, Chin et al. affirmed that a conductive PDA acted as a charge transport bridge by donating plentiful electrons for charge redistribution between ZnO and Ag [77].

There is no critical evidence in their work to support this hypothesis, whereas the following examples may provide clues for later verification. Lin et al. evidenced that the Cu 2p_3/2_ peak in the XPS spectrum shifted to a lower energy level gradually with the sequential addition of PDA and Ag to CuO. This phenomenon revealed that the middle layer of PDA provided a large number of π electrons, which could affect the charge redistribution between Ag and CuO. Raman spectroscopy also showed that this PDA interlayer between the noble metal and semiconductor material could indeed achieve additional Raman scattering enhancement [99].

The thin-layer PDA was an organic semiconductor material that could transfer electrons to Ag NPs to enhance the surface plasmon resonance [100]. In this case, by electrostatic interaction, the positively charged PDA could be coated on the negatively charged ReS_2_ nanoflake, and then the negatively charged Ag NPs could be carried by ReS_2_/PDA that had been converted to a positive charge by PDDA (Figure 5b). Hu et al. found that the intermolecular electron transition from HOMO to LUMO of 6-BAP was not achieved under the excitation of light at 785 nm with low energy. As an energy level coupling platform, composites composed of ReS_2_ and PDA (Figure 5c) reduced the band gap and stimulated efficient PICT. Simultaneously, the proposed process that the electrons were injected into the CB of ReS_2_ from Ag NPs, and that the charges were transferred from the HOMO of 6-BAP to the CB of ReS_2_ to the Fermi level of DA and finally to the LUMO of 6-BAP (Figure 5d) provided a reasonable explanation for the SERS phenomenon.

Nevertheless, this property of PDA cannot only be applied to the design of nano-composite materials with the combination of precious metals and metal semiconductors, and its potential for small molecules and polymers needs to be further investigated. Doping PDA into a semiconducting conjugated polymer would increase electron transfer and reduce the optical bandgap energy to amplify Raman scattering. For example, DA and 5,6-dihydroxyinode, the intermediate in the assembly of PDA, have a similar five-member nitrogen-containing heterocyclic ring with a pyrrole monomer. This phenomenon led to the formation of π-π stacking between PDA and polypyrrole (PPy). In fact, the DA monomers were seen as the better dopant to break the chain of PPy, but they tended to polymerize under the reaction condition with oxidative reagents. It was reasonable to decrease the initial pH and the amount of DA molecules to inhibit the conformation of non-hybridized polymers. By replacing PPy with another conjugated polymer, polyaniline, the same effect could be achieved. Then, Liu et al. added chondroitin sulfate, DA, and pyrrole on the outer layer of SiO_2_ nanospheres to synthesize a novel material for Raman and photoacoustic imaging. Beyond that, plenty of reactive moieties in the molecular construction of PDA provided sites for other functional units to achieve magnetic resonance imaging and targeted therapy [101]. Yan et al. constructed an elaborate photoelectrochemical sensor and proved that PDA could assist charge transfer by electrochemical impedance spectroscopy. The photocurrent was increased after the PDA film was coated on the electrode. This phenomenon further supports the theory that PDA acts as an electron donor, reinforcing the tendency for photogenerated electron–hole pairs to separate [71].

Therefore, in this section, it is found that the sensor with PDA may have the opportunity to realize the goal of the integration of diagnosis and therapeutics, produce a dual-mode sensor with SERS and photoelectric activity, and be employed for bioimaging.

### 4.2. Confining Signal Reporters

The unique functional groups and rough morphology allow PDA to combine quantities of signal molecules easily, which is favorable for sensors that require SERS tags to obtain an external signal for analysis. The dynamic cycle signal amplifying system can also be constructed on the PDA. However, it is worth noticing that the amount of PDA needs to be controlled because the two Raman peaks of PDA are generally considered as background noise and can interfere with the recognition of target peaks [57].

Zhou et al. found that the distribution and quantity of Au NPs on the PDA nanospheres can be modulated by adjusting the mass ratio of HAuCl_4_ and PDA and the reaction temperature, thereby preserving more binding sites for signal molecules [76]. The signal molecules modified with sulfhydryl groups could be chemically bonded to the Au NPs, and the signal stability could be longer lasting after being wrapped with PDA of strong adhesion [102]. Small aromatic thiols are popular candidates for optimal Raman signal reporters, having a single and distinct peak [4]. Directly wrapping the reporter molecules containing thiols on a plasmonic particle with a thin layer is called a self-assembled monolayer (SAM). Li et al. discovered that compared with SAM, the introduction of a thicker PDA film could better utilize the electromagnetic field from a single particle to enhance the intensity of the SERS signal. Thiol groups can covalently bind to DA molecules via the Michael addition reaction. Compared to bare Ag NPs, PDA-coated Ag NPs exhibited an almost 20-fold increase in the optimal amount of 4-nitrobenzenethiol that could be carried. They also tested six other signal molecules, and the results all showed that this variety of three-dimensional volume-active SERS probes deadly confined more reporters and achieved more significant SERS signals than SAM-based SERS tags. Finally, regarding the strength of this design, 4-(phenylethynyl)benzenethiol including alkynyl, having a distinct peak in the signal silence region of biological tissue, was selected to construct probes for tumor imaging [103].

MB was a common, cheap, and low-toxicity dye that could be absorbed on PDA via electrostatic attraction and π-π stacking interaction for both FL and Raman sensing [76]. Other fluorescent substances and their derivatives with strong absorption are also worth examining to generate distinguishable signals for dual-mode sensing [28,104]. Both FL and SERS intensities depend on the distance between molecules and metal particles with enhanced electric and magnetic fields. Unlike SERS, the FL intensity tends to first increase and then decrease as the molecules move away from the metal surface. To avoid the instability of dynamic analysis, a thin dielectric material shell, being chemically and electrically inert, can be inserted between molecules and metal particles to adjust the distance between them [105,106,107]. Compared with Ag nanocubes combined with SiO_2_, Ag nanocubes modified with PDA could trap more Raman reporters through π-π stacking interaction and hydrogen bonding between PDA and R6G, resulting in a more prominent SERS signal [63]. Wu et al. indicated that hydrogen bonding and π-π stacking forces between PDA and the aptamer nucleobases locked R6G tightly in the pores of mesoporous SiO_2_ NPs. Through the collaboration of the acidic environment and strong hydrogen bonding between the target analyte and the aptamer under PDA, PDA was degraded and the signal molecules were released to combine with additional SERS substates to achieve quantitative analysis [108]. PDA-functionalized Au bipyramids can be utilized in conjunction with a dual-mode probe that exhibits both SERS and fluorescent. This probe was a phenylboronic acid-substituted distyryl boron dipyrromethene [109], which enabled the boric acid to interact with the catechol groups of PDA.

After all, a single substrate has a fixed surface area, and the number of reporter molecules it can carry is limited. Therefore, the SERS biosensor modified by PDA may be able to cooperate with some automatic cyclic signal amplification systems. For example, when an aptamer with one signal molecule bound to the target analyte and then was away from the sensing substrate, the complementary nucleotide chain formed a hairpin structure, and another signal molecule at its tail approached the sensing substrate and output the corresponding signal. Usually, better statistical results would be reached by the ratio of two signals rather than a single signal [102,110]. The aptamer that recognized the target analyte was then digested by the exonuclease, allowing the analyte to be released to continue reacting with the sensing substrate [111].

To sum up, modifying the thickness, roughness, and other spatial structures of the PDA layer leads to an increase in the number of binding sites available for signal molecules. Of course, it is also desirable to use the adhesive properties of PDA to bond more components that can be directly connected to signal molecules. Moreover, PDA can isolate signal molecules from the complex environment as an internal standard to output more stable and accurate results. Importantly, the signal molecules could be close to or far from the surface of SERS substrates as expected to enable the Raman signal to be turned on or off, which may be a more promising design. Since the molecular structure of the Raman reporter is not damaged in the process of Raman signal detection, it is advantageous to realize multi-mode sensing with the assistance of multifunctional molecules. Finally, a dynamic cycle signal amplification system is mentioned, which can not only help the sensor to obtain obvious target signals but can also save consumables.

### 4.3. Identify Target Analytes

Most proteins, peptide chains, nucleotides, small molecules, and polymers can bind to PDA through electrostatic attraction, hydrogen bonding, π-π stacking, and cation–π interactions [17]. On the one hand, it was convenient for PDA-smeared materials to conjugate with thiol or amino group modified biomolecules, such as antibody (Ab) [112], nucleic acid [110], and aptamer [47], for specific recognition. Without PDA, the introduction of amino-containing biomolecules required the additional carboxyl groups to be activated by 1-ethyl-3-(3-dimethylaminopropyl) carbodiimide and N-hydroxy succinimide [113]. This synthetic process was tedious. Additionally, several ingredients might not be completely cleaned, which affects the presentation of the Raman peak. The amino group on the Ab could be assembled to the PDA shell by the Michael addition reaction [102]. Through Schiff base reactions, anti-human epidermal growth factor receptor monoclonal Ab was added on the PDA surface to obtain the selectivity of SERS probes [103]. Single-strand nucleotides and proteins could be attached to PDA with numerous functional groups by π-π stacking to shorten the incubation time of the immunoassay [114]. The PDA layer with high density and roughness could hold more Abs and keep them active for a long time. However, it was necessary to find a proper concentration of DA for self-assembly because the active sites of the Ab would be buried by PDA of too high density [115]. On the other hand, a PDA film also makes a contribution to non-specific sensors. 4-carboxyphenylboric acid could be introduced by PDA, and boronic acid could interact with the bacterial diol group of the saccharide [116]. The PDA layer could also use π-π and hydrogen bonds to attach some target molecules directly [63]. Intriguingly, the combination of PDA and a phospholipid bilayer [47] may have promising applications in targeted sensing.

**Figure 6:**
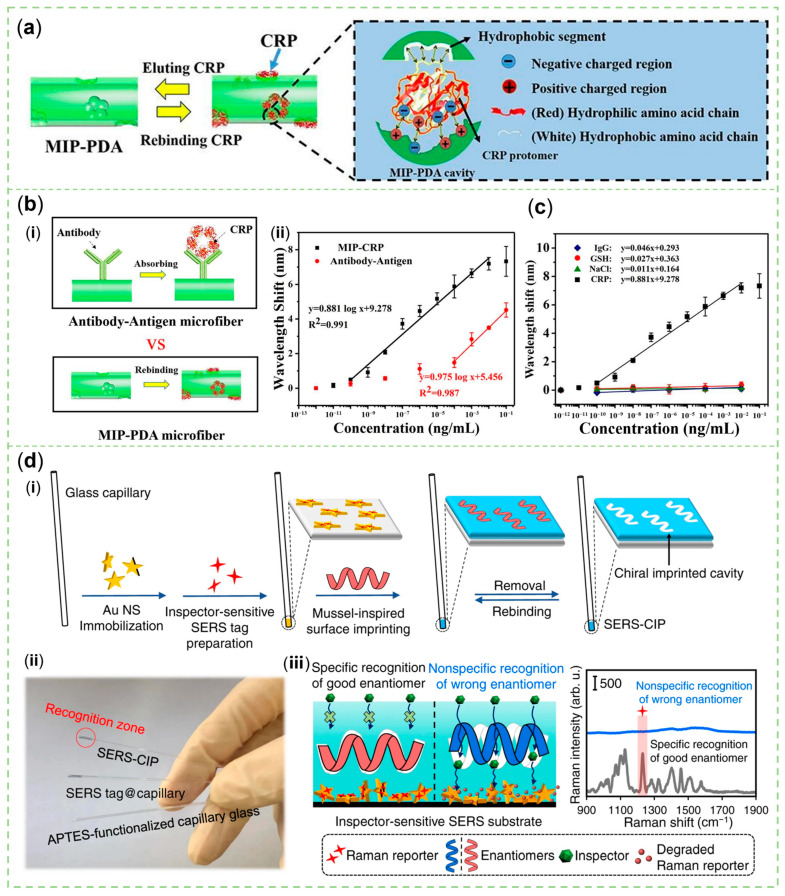
Design and characteristics of SERS sensor related to PDA MIP. (**a**) Diagram of CRP-induced polymerization of PDA. (**b**) (**i**) Schematic of the Ab-antigen microfiber probe functionalization. (**ii**) Linear fitting of Ab-antigen microfiber response to CRP injections. (**c**) Spectral wavelength shifts of MIP-PDA microfiber chemosensor responding to CRP, NaCl, GSH, and IgG at gradient concentrations [49]. (**d**) (**i**) Schematic illustration of the SERS-chiral imprinted platform (CIP) construction. (**ii**) Photo images of SERS-CIP. The recognition zone is illustrated by a red circle. (**iii**) Principle of an “inspector” recognition mechanism implemented on SERS-CIP [120]. Copyrights: all images have been adapted and reproduced with permission from: (**a**–**c**) © 2022 Elsevier B.V.; (**d**) © 2022, Maryam Arabi et al. published by Springer Nature.

In the process of oxidative polymerization of DA for MIP, the target molecule can be used as a template doped in the molecular structure of PDA. After the template is removed, the remaining cavities corresponding to the size, shape, and functional groups of the target can be specifically matched to the target to be recognized. For example, amino and carboxyl groups in peptide chains could link to hydroxyl and amino groups in PDA [117]. Yang et al. synthesized a PDA-MIP-coated SERS sensor with moderate Au NPs that did not mask the recognition site for analyzing three varieties of phthalate plasticizers [118]. To increase the amount of binding sites, this PDA-based MIP could be coated on the SiO_2_ NPs, wherein SiO_2_ NPs were removed by hydrofluoric acid to produce a stable, homogeneously-dispersed, hollow configuration [119]. Nevertheless, in real samples with a lot of interferences, the selectivity of this artificial Ab would be weakened. Morphological and molecular weight analogs of target analytes may occupy sites. Liu et al. used the target biomarker, C-reactive protein (CPR), as the template to mediate the structural rearrangement of (DA)_2_/DHI trimers in the PDA film of MIP-PDA microfiber sensors. The hydrophobic area of CRP was combined with the hydrophobic area of the benzene ring in DA and the negatively charged carboxyl groups on CRPs were attached to the positively charged -NH_2_ or -NH groups on DA and DHI (Figure 6a). Even if the template was finally eluted, this hydrophilic/hydrophobic and charged distribution compatible with the substance to be recognized could be retained to enhance the specificity of the sensor. Eventually, MIP-PDA showed superior recognition ability when compared with an antigen-Ab binding assay (Figure 6b) and under the interference of glutathione (GSH), NaCl, and immunoglobulin G (IgG) (Figure 6c) [49].

The enantiomers of a chiral molecule may exert completely different effects on the health of the body, but they are hard to tell apart. Arabi et al. selected a linear-shaped aminothiol molecule as an inspector for the PDA chiral imprinted cavities coated on SERS nanotags (Figure 6d). The nanotags were composed of gold nanostars (Au NSs) and 3.3′-diethylthiatricarbocyanine iodide (DTTC). The adjustable density and thickness of the PDA layer was a determining factor in changing the permeability of the inspector. Passing through cavities unsuccessfully combined with good enantiomers, qualified inspectors could irreversibly and quickly degrade DTTC to obtain a decreased SERS signal [120]. Moreover, Kong et al. found that the molecular structure of PDA synthesized on the surface of SiO_2_ with a certain chiral characteristic would change accordingly. PDA could discern the corresponding tyrosine and phenylalanine enantiomers because of the identical chirality from a phenethylamine molecule-like unit and π-π stacking interactions from benzene rings [121].

Electrokinetic pre-separation enhanced the concentration of target charge molecules on the AuNP@PDA-MIP surface, and improved the selectivity and sensitivity of SERS sensors. One thing to note is the thickness of the PDA-MIP layer. For the layer, being too thick would reduce the electromagnetic field intensity around AuNPs, negatively affecting SERS performance; meanwhile, if it was too thin, it would be destroyed under a high electric field and lose its specificity [122]. In the meantime, PDA was pH responsive due to amine and phenolic hydroxyl groups [123], and could be negative in the alkaline environment, attracting identified molecules with a positive charge [51].

To enable rapid point-of-care testing in vitro, interfering factors from blood, urine, saliva, and other various biological samples need to be eliminated without pretreating the sample [124]. Undoubtedly, with regard to in vivo testing, this issue necessitates further attention [125]. Raman peaks, like fingerprints, bring convenience to the analysis of results, but the SERS sensors capturing substances non-specifically make the follow-up work extremely tedious. Therefore, SERS biosensors can specifically capture target analytes and cooperate with signal molecules to construct signal enhancement or attenuation mode, especially in the case of no sample pretreatment. Fortunately, PDA can directly chelate metal ions, directly bind small molecules and biomacromolecules, select targets with different charge and chirality, or use recognition elements. At present, PDA is also expected to construct advanced and environmentally friendly MIP.

Essentially, the improvement in the above three functions is far from sufficient for the commercialization and reuse of SERS sensors. Therefore, the fourth part of this review discusses the enhancement of their practicality.

### 4.4. Enhancing the Practicality of Sensors

Here, as can be seen from some cases concerning powder materials, enhanced stability, uniform dispersion, and resistance to contamination can ensure the reliability of test results and can facilitate long-term preservation. To overcome the challenges of uneven distribution, difficult collection, and reuse of powder materials, it is recommended to incorporate medical consumables, such as test paper, cotton swabs, and glass rods, and smart platforms, such as microfluidic devices, microarray, and lateral flow assays, for rapid sample collection and detection. In most experiments, the method of dripping liquid samples on the silicon wafer and then drying them has been adopted to conduct multi-site detection. However, in the actual testing process, the distribution of analytes to be tested is quite uneven, which reduces the repeatability. The hydrophobic treatment of platforms carrying liquid samples can improve this situation. Additionally, the research on two-dimensional PDA membranes with special textures and strong mechanical capacity needs to be accelerated, which can reduce the consumption of raw materials. Fortunately, the biocompatibility and biodegradability of PDA have been extensively researched in therapeutic and sensing applications, providing strong support for its use. However, its problem of long-term preservation after being administrated into the organism remains unsolved. The practicality of SERS sensors can be improved through the implementation of various approaches, as listed in Table 1.

A qualified sensing element must first ensure stable performance in complex practical application environments. Additionally, it cannot have a destructive effect on the targets. Especially in the biological samples, good biocompatibility is highly important. PDA could decrease the biotoxicity of various nanomaterials, which is conducive to the production of biosensors without damaging normal tissues and cells [77,137].

The PDA layer can protect the highly oxidizable metal matrix [128]. Renard et al. synthesized octahedral aluminum nanocrystals coated with a 5–7 nm PDA layer binding to Al_2_O_3_ by catechol groups. PDA functionalization provided them with structural stability in Milli-Q H_2_O for up to two weeks. Since aluminum nanocrystals are extremely unstable in aqueous environments, the common Tris buffer could be replaced by isopropanol and NH_4_OH in the synthesis of PDA [127]. This feature could also extend the shelf life of materials and save the cost of preservation [63].

Bovine serum albumin (BSA) could be attached by the formation of covalent bonding between its amino terminus and the quinone groups in the PDA to block non-specific binding sites [71]. PEG with the thiol or amino group could be loaded on the multifunctional nanomaterials by PDA to obtain the antifouling property [113] and inhibit cell or protein adhesion [15]. Liu et al. found that by remodeling DNA, PEG, and PVP on the outer layer of PDA, the NPs could remain stable, based on electrostatic and steric repulsion, under repeated freeze-thaw cycles and in the harsh environment of high ionic strength, serum, and cell lysate [138].

It was difficult to avoid the problem that the SERS matrix in powder form was unstable, easily aggregated, and not suitable for recycling. The smooth interface of a glass capillary made the test result repeatable due to the uniform coating. In addition, the LOD was low, and the mass production was easy. PDA could be attached to it by covalent and non-covalent bonding, and then loaded with Au NSs [117]. Chang et al. placed the PDA-attached matrix in water, spread silicon or polystyrene (PS) nanospheres with different diameters over the air–water interface, and then discharged the water to obtain a colloidal crystal monolayer, stuck stably and uniformly on the matrix. The versatile application of PDA also enabled the formation of bilayers or trilayers in colloidal crystal. The metal grid, polydimethylsiloxane, polyethylene terephthalate (PET), glass slide, silicon wafer, single mode fiber, and glass capillary could be functionalized by this simple method for a promising SERS application [139].

Fortunately, flexible platforms, such as PET film [75], filter paper [78], or cotton swabs [129], can uniformly disperse the powder over its surface and conform well to the shape of the test object. Li et al. used PDA to assist with the deposition of W_18_O_49_/Ag composites on the hydrophilic modified polyvinylidene difluoride (PVDF) membrane [130]. The selection of an ideal paper with simple Raman spectra as a carrier for the SERS substrate should be considered [128]. Through chemical bonding, PDA could be coated on SiO_2_ nanofibrous membranes prepared by electrostatic spinning and calcination with strong thermal stability, no fracture after folding, and no SERS peaks [131]. PDA could be firmly attached to the conical poly (lactic-*co*-glycolic acid) microneedles. The catechol moieties in PDA were combined with Ca^2+^ and then electrostatically attracted PO_4_^3−^ to obtain randomly-directed, petal-like hydroxyapatite which provided an interface for massive Au nucleation and growth. This device has high mechanical stability, biocompatibility, and detection capability to be inserted into the epidermis and dermis for biosensing [126]. The amino and catechol moieties of PDA facilitated its adhesion to the non-woven fabric with high permeability and a large surface area, followed by reduction of Ag ions resulting in a strong SERS signal [132]. PDA-decorated polyurethane sponges retained high porosity, allowing rapid swab sampling, and could internally generate photon diffusion due to the flexible, hierarchical, and three-dimensional interconnected framework [133].

**Figure 7:**
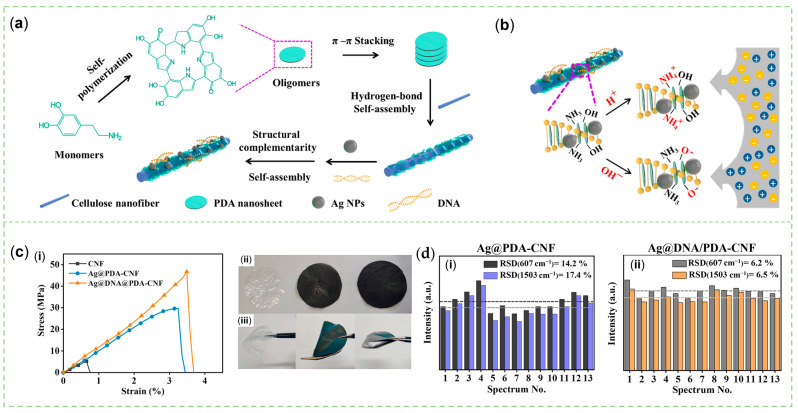
The advantages from the DNA-modified molecular structure of PDA for the SERS sensor. (**a**) Formation mechanism and structure diagram of the Ag@DNA/PDA-cellulose nanofibers (CNF). (**b**) Schematic showing the charge selectivity of the Ag@DNA/PDA-CNF film to solution molecules in acidic and alkaline media. (**c**) (**i**) Stress–strain curves of the samples under a dry condition. Photograph of each membrane (**ii**) in the natural state and (**iii**) when it was folded by tweezers. (**d**) SERS spectra of the R6G solution of 10^−5^ M were obtained by randomly collecting 13 points on Ag@PDA-CNF and Ag@DNA/PDA-CNF. The corresponding SERS intensities distributed at 607 and 1503 cm^−1^: (**i**) Ag@PDA-CNF and (**ii**) Ag@DNA/PDA-CNF [51]. Copyrights: all images have been adapted and reproduced with permission from: (**a**–**d**) © 2021, American Chemical Society.

Xu et al. discovered that the presence of hydrogen bonds, van der Waals forces, and electrostatic attraction helped the flake PDA tetramers adhere to the rod-like CNF. The PDA NSs were rearranged by the introduction of DNA molecules with double helix structures through π-π conjugate bindings between DNA bases and PDA oligomers (Figure 7a). This ordered configuration made the nanofibers possess charge selectivity (Figure 7b), and stronger mechanical properties (Figure 7c), and Ag NPs attached to them were evenly distributed, resulting in a wide range of hot spots. Using R6G as the Raman probe, smaller relative standard deviations at 607 and 1503 cm^−1^ (Figure 7d) were attributed to a more uniform assignment of hot spots of the Ag@DNA/PDA-CNF compared to the Ag@PDA-CNF [51]. This result indicated that the participation of DNA in the polymerization process of PDA could improve the repeatability of SERS sensors. The use of bio-derived materials to produce wearable flexible sensors adapted to human tissue curves has been extensively investigated [14]. Hence, PDA could be combined with CNF, cellulose nanocrystals, SF, carboxymethyl cellulose, and other sustainable materials to prepare intelligent SERS sensors in the form of controllably degradable biomedical implants or air-permeable, easily-detachable, adhesive artificial skin for in situ diagnosis and on-demand treatment.

**Figure 8:**
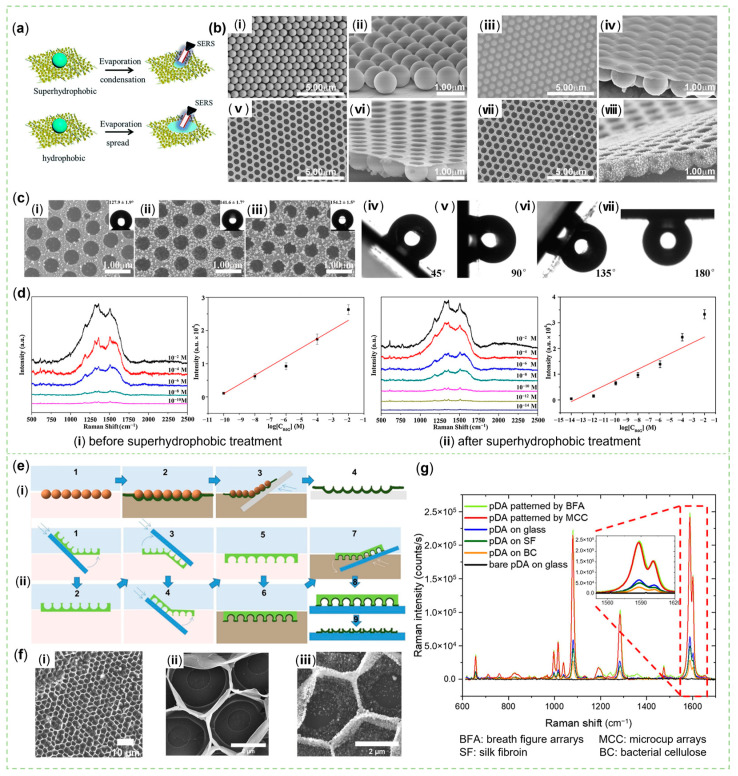
The hydrophobic PDA films for SERS sensors. (**a**) Schematic illustration for the evaporating and spreading process of the droplet [142]. (**b**) Scanning electron microscopy (SEM) images of (**i**,**ii**) monolayer PS colloidal crystals; (**iii**,**iv**) PS-PDA colloidal monolayer film; (**v**,**vi**) PDA microbowl array film and (**vii**,**viii**) PDA-Ag microbowl array substrate. SEM images were observed from different directions: (**i**,**iii**,**v**,**vii**) from the top view; (**ii**,**iv**,**vi**,**viii**) from the side view. (**c**) SEM images of PDA-Ag array substrates prepared using different concentrations of [Ag (NH_3_)_2_]^+^ ions: (**i**) 1.2 × 10^−2^ M, (**ii**) 2.4 × 10^−2^ M, and (**iii**) 4.8 × 10^−2^ M and modified with *1H*, *1H*, *2H*, *2H*- perfluorodecanethiol (PFDT). Insets: the corresponding water contact angle measurement; (**iv**–**vii**) photographs showing a water drop on the substrate (**iii**) with tilted angles from 45° to 180°. (**d**) Comparison of the detection range of the PDA-Ag array between before (**i**) and after (**ii**) the superhydrophobic treatment [134]. (**e**) Fabrication scheme of patterned PDA surfaces by using closely packed colloidal spheres (**i**) and breath figure arrays (**ii**) as templates. (**f**) SEM images of PDA film patterned with breath figure arrays (**i**,**ii**) and further decorated with Ag NPs (**iii**). (**g**) SERS activity of PDA films with or without special patterns [135]. Copyrights: all images have been adapted and reproduced with permission from: (**a**) © The Royal Society of Chemistry 2017; (**b**–**d**) © 2017 Elsevier B.V.; (**e**–**g**) © 2022 Elsevier B.V.

As the volatile solvent evaporates from sessile droplets, capillary flows caused by contact line pinning transport non-volatile solutes to the contact line, resulting in the coffee-ring effect (CRE). Suppressing or exploiting the CRE so that the solute is concentrated and uniformly distributed at a central point or on the contact line at the outer edge [140,141] is also a matter of concern in constructing a SERS detection device with good reproducibility. The sample preparation platform with hydrophobic properties could condense the solution droplet and distribute the effective analytes relatively uniformly in a small area after drying (Figure 8a). For SERS sensors, which conducted detection at multiple points on the sample and then obtained the average value, more valuable repeatability and smaller error could be acquired [142,143]. Particularly, PDA films with a variety of patterns also showed better hydrophobicity with a positive effect on SERS activity [135]. It was generally considered that the introduction of low surface energy chemicals and the construction of a rough surface with micro-, nano-, and hierarchical structures were two ways to obtain hydrophobicity [144]. Some hydrophobic alkanes with sulfhydryl groups, such as n-dodecyl mercaptan and hexadecanethiol, could be easily imported into biosensing materials through the Michael addition reaction with PDA [145]. Dong et al. modified PDA-decorated cellulose filter paper with PFDT to gain more sensitive SERS results than with non-hydrophobic substrates [136]. With respect to the rough surface with special structures formed by PDA, Shang et al. used trichloromethane to remove monodisperse PS photonic crystal nanospheres to prepare a superhydrophobic PDA microbowl array (Figure 8b). The array not only had hydrophobicity due to PFDT and roughness from Ag NPs (Figure 8c(ⅰ–ⅲ)) but also had high water adhesion (Figure 8c(ⅳ–ⅵ)) both to constrain the sample well and to obtain a lower LOD (Figure 8d). The PDA-Ag microbowl array maintained good hydrophobicity and SERS activity even under a harsh environment [134]. In developing this kind of sensor, two contact states, the Wenzel state and Cassie state, between the substrate with a special texture and the droplet need to be explored [146]. Clearly, Yu et al. found that although honeycomb porous and pincushion-like PDA films had high hydrophobicity, they had completely different water adhesion [147]. It is necessary to point out that a PDA film could be merely fabricated at the air/water interface in the Tris buffer containing DA. Making good use of this feature, Kozma et al. placed different sacrificed templates to float on the liquid surface in order to pattern the PDA film. Microcup arrays, an inverse opal structure with 500 nm voids, were obtained by closely packed PS microspheres (Figure 8e(i)), and honeycomb structures were made by breath figure arrays (Figure 8e(ii),f). Furthermore, the resulting film, transferred to a piece of glass, was annealed at 100 °C for 10 min to enhance its mechanical properties. Subsequently, Au NPs were attached by reduction to confer SERS ability on the PDA film. Such SERS substrates with a large surface area, hydrophobicity, and new photonic features showed brilliant SERS performance (Figure 8g). They also reported that bacterial cellulose and silk fibroin, some natural polymers, could support them for promising applications [135].

The mechanical properties of PDA, such as compressive strength, adhesive strength, and elastic modulus, will be the hot spots for practical applications in the future [17]. Coy et al. prepared free-standing and transferable PDA thin films with high hardness and elasticity [148]. The novel semi-permeable PDA film, similar to the cell membrane, could incorporate molecules with specific biological functions for the separation of target analytes [149]. However, the long-term accumulation of SERS tags functionalized by PDA in the livers of mice may lead to side effects in the body, which needs to be carefully considered [84].

## 5. Biomedical Application

In the past, multiple studies have proved that the realization of sensing and imaging through innovative SERS substances was conducive to monitoring diverse diseases and evaluating post-treatment conditions [30,150,151]. Nonetheless, there is still a long way to go to produce biosensors that can meet the budget and are suitable for a variety of site applications to achieve portable, field-deployable, accurate, rapid, and affordable point-of-care diagnostics [30,124,152].

PDA-modified materials cooperating with proteins, enzymes, peptides, nucleotides, polysaccharides, lipids, and living cells have attracted much attention in the field of biomedicine. PDA is usually able to retain the biological activity of the biomolecules. Particularly, after in vitro and in vivo tests, valuable biocompatibility can be demonstrated at the level of the tissue, cell, protein, and gene. They are beneficial for cell adhesion and growth, avoid damage to mitochondria, reduce the production of reactive oxygen species, and resist inflammation and senescence [47]. As the basic unit of the human body, cells can produce proteins, nucleic acids, lipids, reactive oxygen species, and other biomolecules. The specific abnormal expression of certain biomolecules can be a sign of serious disease [153]. Biosensors are often prepared with the goal of distinguishing and monitoring the type and level of this substance for prevention, diagnosis, and follow-up. The following section discusses the medical diagnostic applications of related materials in SERS biosensing as reported in recent research.

### 5.1. Detection of Nucleic Acid

Nucleic acids, containing DNAs and RNAs, as promising biomarkers of several diseases have complex molecular structures that can be adhered to PDA film easily to endow SERS materials with the ability of biorecognition. There were some excellent instances, as follows, offering instructive advice for designing such biosensors.

**Figure 9:**
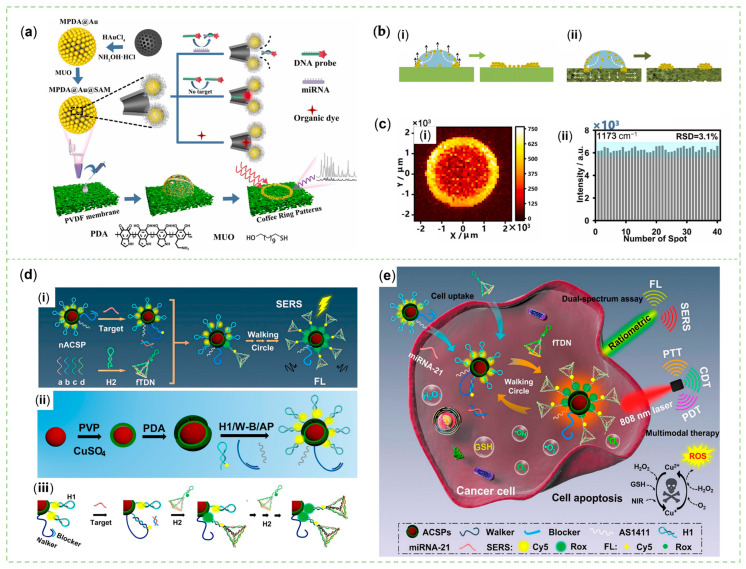
SERS biosensors for nucleic acid detection facilitated by PDA. (**a**) Schematic representation of the construction of SERS sensing devices based on two-tier host–guest architectures and regioselective enrichment effect. (**b**) Schematic illustrations for the process of forming a coffee ring pattern on the nonporous support-glass sheet (**i**) and porous support-PVDF membrane (**ii**). (**c**) SERS mapping results when using PVDF membrane (**i**); the bar chart for the peak intensity on PVDF membrane (**ii**) [158]. (**d**) (**i**–**iii**) Design and detailed working principle of the fTDN (fuel DNA-conjugated tetrahedral DNA nanostructures)-assisted DNA walking nanomachine for simultaneous ratiometric SERS-FL assay of microRNA (miRNA)-21. (**e**) Illustration of the developed nanodevice for miRNA detection and imaging in living cells and Au@Cu_2−x_S NPs (ACSPs)-mediated multimodal synergistic therapy [110]. Copyrights: all images have been adapted and reproduced with permission from: (**a**–**c**) © 2022 Elsevier B.V.; (**d**,**e**) © 2021 American Chemical Society.

Obtaining a good balance between constructing a lot of hot spots in different dimensions [154] and improving the quantitative capacity is not an easy task, especially for actual sample testing [155]. Molecules entering the region of SERS-active materials with hot spots show an anomalously significant signal [156]. Therefore, it is a top priority to obtain all the target analytes in the sample into the signal enhancement, and to eliminate the disruptors. This purpose can be attained by porous enclosures that restrict the proximity of distractors [155] and materials that achieve anti-fouling effects through hydration and steric hindrance [157]. To control analyte–particle and particle–particle distances in the detection matrix and improve the stability and repeatability of the sensing, Zhou et al., designed host–guest nanosensor particles (MPDA@Au-SAM) (Figure 9a) that were uniformly assembled into a coffee ring pattern on the surface of a porous PVDF film. In their work, mesoporous PDA (MPDA) NPs with sizes of 150 ± 20 nm and an average pore size of 35 nm were synthesized for the in situ reduction of chloroauric acid. At the optimized HAuCl_4_/MPDA weight ratio of 2.4, largely enriched and uniformly distributed Au NPs could be installed on the outer surface of MPDA. Additionally, then, a SAM of 11-Mercapto-1-undecanol was covered on AuNPs by Au-S bonds. As a result, through the strong π-π stacking interaction of PDA and the low affinity of SAM to aromatic molecules, this nanomaterial could select and enrich SERS active analytes into the pores of MPDA. Moreover, porous materials with a larger surface area could immobilize plentiful plasmonic particles and analytes. In particular, the powerful FL quenching effect of PDA was also used to highlight the Raman signal when they employed a Cy5-labeled DNA probe to recognize miRNA-21 of a lower concentration. The permeable porous platform was utilized to enhance the CRE (Figure 9b) to obtain a lower relative standard deviation (Figure 9c) [158].

The invention of sensors that meet the needs of multi-mode sensing and the integration of diagnosis and treatment is the future trend, which improves the reliability of diagnostic results and is in line with the concept of sustainable development. Zhai et al. produced a SERS/EC sensor, embracing the sensitivity of SERS sensing and the reproducibility of electrochemical sensing. When gastric cancer-related miRNA-106a was present in the sample solution, a sandwich structure was constructed by combining MPA@MB-P2 and the Ag NRs array electrode through the linkage between miR-106a and ssDNAs P1 and P2. Consequently, MB molecules were loaded on the MPA localized in the gap and a featured peak at 1622 cm^−1^ with enhanced intensity could be chosen for quantitative analysis [159]. In their previous research, the MPA could kill *Staphylococcus aureus*, consisting of MoS_2_ NSs, PDA, and Ag NPs. The PDA film coated on the MoS_2_ could be synthesized via a microwave in ten minutes. Ag^+^ ions were absorbed by various nitrogen-containing and phenolic groups of PDA, and then reduced by glucose to form 5 nm particles uniformly distributed on the NSs [160].

Unfortunately, the one-to-one binding of the biometric element to the target nucleic acid strand compromised the sensitivity of the biosensor. However, the implementation of dynamic DNA nanomechanics results in a reduction of the LOD and an expansion of the linear range. For example, using a PDA layer covered on the ACSPs, He et al. were inspired by the dynamic DNA walking nanomachine and constructed a stable signal amplification system by quinone groups to link the sulfhydryl group modified on the nucleic acid molecule. When the target miRNA was present, a DNA walker (Figure 9d) binding the blocker was released, unfolding a hairpin-shaped Cy5-labeled DNA strand H1 on the particle, catalyzing its interaction with the corresponding ROX-labeled H2 strand of a DNA tetramer outside the particle. After the completion of this automatic cycle, the NP was filled with H1–H2 complexes. Additionally, Cy5, initially located close to the surface of the particle and exhibiting strong SERS and weak FL emission, ultimately migrated away from the surface resulting in enhanced FL and diminished SERS response. Conversely, ROX showed the opposite trend. This phenomenon was attributed to the surface plasmon resonance of the metal substrate and the FL quenching ability of PDA for calculating the SERS and FL signal ratio. Moreover, the AS1411 aptamer could be added by PDA to make it easier for ACSPs to enter the diseased cells. The photothermal conversion ability of PDA could also work in cancer therapy (Figure 9e) [110]. Jiang et al. demonstrated that the cooperation of Au@PDA@Ag NPs with 4-mercaptobenzoic acid (4-MBA), magnetic NPs (MNPs), and a re-circulating enzyme amplification system could detect tumor genes. A kind of hairpin DNA with three regions could coexist with the bridging DNA and nicking endonuclease in the absence of target RNA. The 5′ end of the bridging DNA hybridized with aptamer S_1_ on the MNPs. Additionally, the remaining oligonucleotide chain at the 3′ end was related to PDA on the Au@PDA@Ag SERS tag by π-π bonding. Finally, they were all separated magnetically from the supernatant. However, when miRNA-31 was present, the hairpin DNA unfolded, and the target could be fixed on Region Ⅰ. The bridging DNA bound to Region Ⅱ and Region Ⅲ of the hairpin DNA could be cleaved by the nicking enzyme to obtain the S_1_ complementary strand. Importantly, the resulting duplex on the MNPs would not react with PDA to make the SERS tags stay in the supernatant. Next, the released Region Ⅱ and Region Ⅲ were further hybridized with the newly introduced bridging DNA in a cyclic manner to achieve signal enlargement. Consequently, quantitative results were obtained according to the amount of bridging DNA and the SERS intensity of the supernatant [114]. Wu et al. designed a recyclable biosensor to identify miRNA 155, including a magnetic core-shell material constructed with the assistance of PDA, which was biocompatible and highly dispersed, and a signal amplification system that focused on enzymatic DNA digestion [161].

In summary, PDA have been mainly used in the preparation of biosensors for monitoring nucleic acids to reflect the health status by loading probes. Based on the above cases, nucleotide chains with bases and signal molecules can be linked by PDA to enter or leave the electromagnetic field.

Based on various designs, including colloidal solutions and three intelligent platforms, Table 2 summarizes the data of PDA-based SERS biosensors applied in the biomedical field.

### 5.2. Detection of Protein

Abs, peptide sequences, and aptamers are commonly used to recognize protein and are easily captured by PDA. Additionally, interestingly, MIP synthesized by PDA further simplifies recognition components and can be reused for sustainability. In this part, relevant research cases are classified into four sections, powder reagents, LIFA, microarray, and microfluidic platform.

Lu et al., synthesized PDR with a loose surface and functional groups for loading Au NPs. The PDR@Au NPs with SCCA monoclonal Ab cooperated with the hollow Au nanocages with 4-MBA and SCCA polyclonal Ab to recognize SCCA. The material still had good sensitivity and reproducibility when applied to clinical blood samples from patients with cervical cancer and cervical intraepithelial neoplasia, and healthy subjects [162]. Magnetic ingredients can facilitate the separation of powered particles dispersed in the sample, enhance the sensitivity of detection, and recycle the active sensors. Achadu et al. inserted semiconducting oxides, plasmonic metals, and magnetic materials into a hollow and porous PDA nanoskeleton to synthesize mag-MoO_3_-PDA@Au nanospheres with the tunable charge-transfer and LSPR effect via magnetic actuation. 4-MBA with a higher enhancement factor and PICT potential was designated as the Raman reporter attached to Au NPs. Angiotensin-converting enzyme 2 was the capturing agent for the SARS-CoV-2 spike protein. In the detection process, the immunocomplexes with a sandwich type could be enriched and extracted simply by an external magnet from PBS and whole-cell lysate media [68].

**Figure 10:**
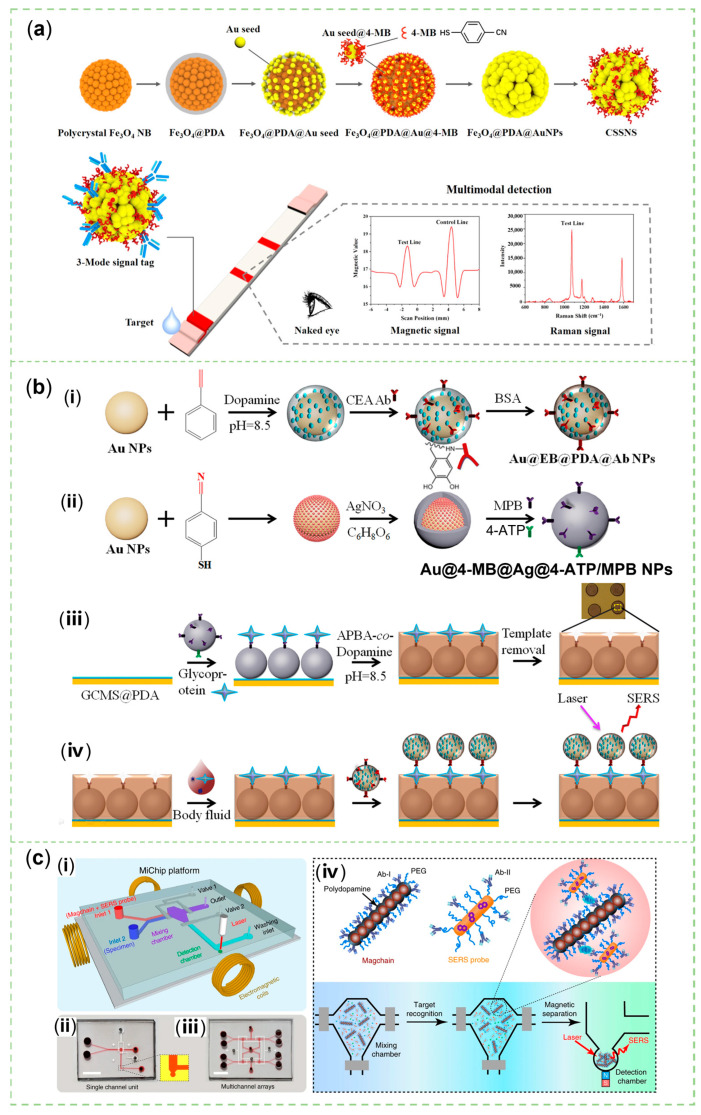
PDA-containing materials cooperated with diverse smart platforms for protein monitoring. (**a**) Schematic illustration of core-shell-shell nanosunflowers (CSSNS) preparation and the multimodal detection of HCG using CSSNS-based lateral flow immunoassay (LFIA) [166]. (**b**) The fabrication process of PDA-encapsulated SERS probes. Components of the (**i**) Au@EB@PDA@Ab NPs and (**ii**) Au@4-mercaptobenzonitrile (4-MB) @Ag@4-ATP/MPB NPs. (**iii**) Synthetic route of preparing boronated affinity-based glycoprotein imprinted arrays. (**iv**) The MIP-based SERS approach for the detection of glycoprotein [102]. (**c**) Design of the magnetic nanochain integrated MiChip. (**i**) Schematic diagram of the MiChip assay platform. (**ii**,**iii**) Photographs of the MiChip: single channel unit (**ii**) and multichannel arrays (**iii**). The microchannel was filled with a red dye for better visualization. Scale bar: 0.5 cm. (**iv**) The MiChip assay for the detection of biomarkers. The specimen, Ab-conjugated magnetic nanochains and SERS probes are mixed in the mixing chamber. The targets of interest in the sample are recognized by the Abs on the Magchains and the SERS probes to form sandwich immune complexes. The immune complexes are then isolated into the detection chamber and subjected to Raman spectroscopic detection [113]. Copyrights: all images have been adapted and reproduced with permission from: (**a**) © 2021 American Chemical Society; (**b**) © 2019 Elsevier B.V.; (**c**) © 2018, Qirong Xiong et al. published by Springer Nature.

A variety of flexible and intelligent devices are needed to lift the usefulness of biosensors. Xia et al. utilized PDA nanospheres to carry Ag NPs and be loaded with 4-ATP and SCCA, or 5,5′-dithiobis-(2-nitrobenzoic acid) and cancer antigen 125. Combined with LFIA, the high precision of SERS detection results in clinical samples showed the potential for the diagnosis of cancer [167]. Exploring the potential of various properties of nanomaterials for multimode detection is conducive to strengthening the reliability of detection results. Liu et al. designed CSSNS combined with LFIA to detect the level of HCG through three signals (Figure 10a), namely color, magnetic, and Raman, for the rapid, accurate, and convenient diagnosis of malignant trophoblastic carcinoma. The dark brown Fe_3_O_4_ nanobeads were hydrophilic and were used as the core for PDA coating. PDA could bind Au^3+^ by amino groups and reduce Au^3+^ to Au seeds by catechol groups, providing an interface for 4-MB. 4-MB also showed reducibility in the formation of Au NPs. The intensity of the 4-MB Raman peak was related to its amount, but it should not be superfluous to occupy the sites for Ab in order to ultimately weaken the SERS effect. After layer-upon-layer modification, polycrystalline Fe_3_O_4_ nanobeads still had high magnetic strength. An ideal diagnostic effect could be obtained by such a sophisticated formulation [166].

The adhesion of PDA has shown an extraordinary effect on constructing arrays for catching target analysis. Wang et al. used the adhesiveness of PDA to arrange PS microspheres on the silicon wafer one by one. Optical confinement from the microsphere’s cavity and the electromagnetic field from Au NPs were beneficial for improving SERS activity. Ab-antigen-Ab sandwich immunocomplexes brought SERS reporters near the chip for a quantitative analysis of the two signature proteins, cTn Ⅰ and creatine kinase isoenzyme MB, that could monitor cardiovascular disease [163]. Huang et al. combined a PDA self-polymerized glass chip possessing albumin Ab with Ab-modified bimetallic SERS tags doped with 4-MPY to detect albumin in urine. The intensity of the peak at 1096 cm^−1^ was of great significance for monitoring the prognosis of cardiovascular disease and diabetic nephropathy [164]. Abundant exosomes derived from cancer cells are continuously released into body fluids, including serum, plasma, and urine. These exosomes play a crucial role in promoting immunosuppression, angiogenesis, cell migration, and invasion during the occurrence and progression of cancer. Carrying information about the tumor microenvironment, they are reliable biomarkers for non-invasive early cancer diagnosis, and the evaluation of therapeutic efficacy. A large number of characteristic proteins on the surface of exosomes are ideal sites for biometrics [168]. Li et al. chose the optimum Ab to capture exosomes by a specific protein expressed on their membranes for the early diagnosis, clinical staging, and tumor metastasis monitoring of pancreatic cancer. A PDA-coated glass chip could easily bind to a variety of Abs. The chip modified by PDA and an Ab was homogeneous and hydrophilic. As a result, the uniform distribution of SERS labels could be achieved without CRE, after the solvent evaporated from the sample on the chip [115].

The selectivity of the PDA-MIP SERS substrate has been fully described above. The following research work will reveal its great potential in biomedical detection. Arabi et al. produced a cheap sensor that could specifically identify four kinds of proteins for quantitative analysis with a SERS signal-reduction mode. A glass capillary coated with Au NSs and a PDA-MIP was immersed in the sample without pretreatment. After the target protein occupied the cavities, a Coomassie brilliant blue G-250 staining step blocked empty pockets between peptide chains so that DTTC could not approach Au NSs [117]. Li et al. immobilized a glycoprotein template in a polymer skeleton containing boric acid and then produced PDA-MIP on it to identify a variety of glycoproteins playing an important regulatory role in metabolism [165]. Significantly, the combination of MIP and Ab may elevate the sensitivity of biosensors. Lin et al. incorporated 4-MB as an internal standard within the gap of Au and Ag bimetal SERS labels to fabricate core-molecule-shell-molecule (CMSM) NPs. A circular array was formed on the modified GCMS through the Michael addition reaction between 4-ATP and PDA (Figure 10b). Then, on the array, CEA glycoproteins were used as the template to form a PDA-MIP layer with appropriate thickness and were eluted cleanly. There were also MPB ligands on CMSM NPs which could covalently and reversibly capture the cis-dihydroxyl of glycoprotein from CEA by boric acid. CEA could still bind to the Ab on PDA-coated Au NPs with EB. The EB molecule had a sharp band at 2024 cm^−1^ for the alkynyl group, and the 4-MB molecule had a narrow band at 2224 cm^−1^ for the cyan croup. Both peaks were located in a Raman silent region, avoiding the interference of fingerprint peaks from endogenous molecules. The ratio of featured SERS peaks of 4-MB and EB had a stronger linear relationship with the content of CEA [102].

Xiong et al. observed the formation of chain-like structures of PDA and Fe_3_O_4_ NPs in an alkaline environment. Notably, its length was increased by lengthening the magnetic stirring time. The shorter chain could be obtained by ultrasonic treatment. By increasing the initial DA concentration, a wider chain was obtained. Stirring bars with the right length and width should be synthesized to achieve the highest rotating speed and the most obvious convection to accelerate mixing in a corresponding rotating magnetic field. The combination of the bioconjugated magnetic nanochains with the MiChip enabled full contact and identification of the target analytes by stirring. Most importantly, it was able to facilitate the reuse of this system without blocking its channels. Finally, Au NRs with different Raman reporters were added to form a sandwich immune recognition suit with nanochains to detect various tumor markers and bacteria in saliva (Figure 10c). This new sensor gained excellent results rapidly, easily, sensitively, and in an environmentally friendly way compared to ELISA and bacterial culture methods commonly applied in the clinic [113].

Obviously, for high sensitivity, selectivity, repeatability, and recyclability, the functions of PDA in the invention of novel and smart biosensors cannot be limited to introducing elements for biological recognition, but all aspects of the performance mentioned in the guideline for designing SERS substrate should be incorporated into our train of thought.

### 5.3. Detection of Other Biological Indexes

Here are some examples of the non-specific recognition of bacteria. Wang et al. captured and magnetically separated bacteria bound to boronic acid-modified and PDA-stabilized SERS labels by IgG@Fe_3_O_4_. Then, through principal component analysis and hierarchical cluster analysis, five pathogenic bacteria can be classified and quantified with a LOD value of 10 CFU mL^−1^ [77]. Wan et al. determined the optimal amount of Ag NPs deposited on SiO_2_ nanofibers for effective differentiation and eradication of diverse bacteria, while the amino and hydroxyl groups in PDA immobilized Ag^+^. The components such as tyrosine, adenine, guanine, saturated lipid, and amide Ⅱ of proteins in bacteria had Raman peaks, which facilitated the label-free SERS discrimination of Gram-negative and positive bacteria [96]. Hong et al. utilized PDA to facilitate the anisotropic growth of Au on Fe_3_O_4_ clusters, resulting in the formation of short microvilli and ultimately achieving a three-dimensional leukocyte-shaped structure. The large surface area of Fe_3_O_4_ microvilli could capture and isolate bacteria for label-free SERS analysis. Moreover, the material to achieve high photothermal conversion under near-infrared light could inhibit Gram-negative and positive, anaerobic, drug-resistant pathogens [169].

Reactive oxygen species consist of the hydroxyl radical (•OH), superoxide (O_2_^−^), and hydrogen peroxide (H_2_O_2_). They can cause a higher level of oxidative stress at organismal, cellular, and molecular levels and have a close relationship with abnormal metabolic activity and mitochondrial function. Thus, they can act as diagnostic markers for various diseases [170,171,172,173]. A kind of chemistry-based SERS nanoprobe with a core-satellite structure assembled by PDA could differentiate the cancerous cells and monitor the autophagy process in living cells. Fe(IV)=O could be obtained with an evident change in SERS intensity at 1386 cm^−1^ through the reaction between reactive oxygen species and the six-coordinated Fe(III)-OH_2_ of myoglobin. The amine groups of myoglobin were proven to be carried by the Michael addition [174].

## 6. Conclusions and Perspectives

This review mainly discussed the application potential of PDA, a biomimetic polymer with rich functions, in the design of new, sensitive, and specific SERS sensors. PDA is considered an eco-friendly material due to its reliable biocompatibility and biodegradability, as well as its simple synthesis process that does not require energy-intensive equipment. Furthermore, the PDA-based SERS sensor finally obtained also had the possibility of reuse. Therefore, the combination of the two was selected to increase the possibility of preparing a biosensor that could be sustainably used. Using the various advantages of PDA to prepare SERS sensors for food safety and environmental protection has received a lot of attention and achieved good research results, such as for the detection of pesticide residues [175], and toxins [108] on the surface of fruits and vegetables, harmful substances in water, etc. However, there is a paucity of research studies conducted on PDA-based SERS sensors within medical field.

The SERS assay has some disadvantages that can be solved by combination with other analytic methods to increase confidence. For instance, the laser excitation region is small so multiple spots should be randomly selected on the sample to be tested for SERS characterization to calculate the average value. However, the SERS signal results obtained at each site can vary greatly, which leads to worse reproducibility. Thus, the electrochemical sensor with relatively stable signals from the entire electrode can be introduced to compensate for this shortcoming [159]. PDA also demonstrated the ability to amplify photoacoustic signals [137]. Integrated diagnostic and therapeutic materials can provide precise location and dosage guidance, minimizing drug waste while optimizing treatment outcomes. PDA was not only a material with a good photothermal treatment effect, but also could carry multiple chemical drugs for chemodynamical treatment [137]. In the field of tissue regeneration engineering, amyloid fibers were employed as catalysts and peptide nanostructures were utilized to direct the polymerization of DA. Subsequently, boric acid was bound to catechol groups in PDA for pH-responsive properties [176]. In the molecule of DA, there are four sites on amino, alkyl, and aromatic rings that can be chemically derived. DA derivatives and their analogues with better surface modification abilities can learn from the cases of PDA in order to obtain more practical SERS sensors. For example, plant-derived polyphenols exhibited swift metal–ligand coordination kinetics for the formation of metal-polyphenol network coatings, in addition to possessing antibacterial and antioxidant biological properties [17].

Through this review, we not only demonstrate the convenience of PDA in fabricating SERS biosensors, but also provide insights into further developments of multimodal sensors and theranostic applications. We believe that this study will be of interest to diverse researchers in the interdisciplinary fields of nanomedicine, biosensing, organic chemistry, and materials engineering, and we hope that it will rapidly stimulate more applied research in this area.

## Figures and Tables

**Table 1:** Various approaches were used to enhance the practicality of SERS-sensors.

Properties	Materials	Ref.
biocompatibility	Ag/PDA/ZnO-filter paper	[77]
	Au-HA *-PDA-PLGA *-microneedles	[126]
inoxidizability	Ag nanocubes@PDA	[63]
	Fe/Fe_4_N@Pd/C	[79]
	PDA@Ag-anti-cTn Ⅰ *	[80]
	Al nanocrystals@PDA	[127]
	Al NPs@PDA on cellulose paper	[128]
antifouling property	Au@EB *@PDA@Ab@BSA	[102]
	Magchains *@PDA@PEG@Ab	[113]
uniformity	Capillary glass@Au NSs@MIP	[117]
flexibility and uniformity	PET/PDA/ZnO/Ag	[75]
	filter paper@PDA@Ag NPs	[78]
	cotton swab@PDA@Ag NPs	[129]
	W_18_O_49_@Ag/PDA@PVDF-MIP membranes	[130]
	Ag@PDA@SiO_2_ nanofibrous membranes	[131]
	non-woven fabrics@PDA@Ag NPs	[132]
	polyurethane sponge@PDA@Ag NPs	[133]
mechanical stability and uniformity	Ag@DNA/PDA-CNF	[51]
	Au-HA-PDA-PLGA-microneedles	[126]
hydrophobic or superhydrophobic	PDA-Ag microbowl array	[134]
	PDA film patterned by microcup or breath figure arrays	[135]
	cellulose filter paper@PDA@Ag NPs	[136]

* HA: hydroxyapatite; PLGA: poly (lactic-co-glycolic acid); cTn Ⅰ: cardiac troponin Ⅰ; EB: ethynylbenzene; Magchains: magnetic nanochians.

**Table 2:** Data on SERS biosensors with participation of PDA.

Different Design	Material	Method	Targets	Linear Detection Range	LOD	Ref.
**Colloidal** **solutions**	ACE2 *-mag-MoO_3_-PDA@Au-4-MBA	SERS	SARS-CoV-2 spike protein	10 fg mL^−1^–1 ng mL^−1^	4.5 fg mL^−1^ (in PBS)	[68]
					9.7 fg mL^−1^ (in whole-cell lysate media)	
	PDA@Ag-anti-cTn Ⅰ *	SERS	cTn Ⅰ	-	0.01 ng mL^−1^ (in PBS)	[80]
				-	0.025 ng mL^−1^ (in human serum)	
	Au@Cu_2−x_S@PDA	FL	miRNA-21	1 pM–10 nM	0.11 pM	[110]
		SERS		10 aM–1 nM (in vitro)	4.95 aM	
				0.29 fM–9.30 pM (in living cells)	0.11 fM	
	Au@PDA@Ag	SERS	miRNA-31	0.6–1.8 fM	0.2 fM	[114]
	Fe_3_O_4_@PDA/Pt-TB/S1 and AuNFs-modified S2	SERS	miRNA 155	1 fM–10 μM	0.28 fM	[161]
	PDR^*^@Au NPs and Au nanocages@4-MBA	SERS	SCCA	10 pg mL^−1^–1 μg mL^−1^	7.16 pg mL^−1^ (in PBS)	[162]
					8.03 pg mL^−1^ (in peripheral blood)	
**Modified chips**	Au@EB *@PDA@Ab NPs and GCMS *@PDA-Au@MB@Ag@4-ATP */MPB * NPs-MIP circular array	SERS	CEA *	0.1 pg mL^−1^–10 μg mL^−1^	0.064 pg mL^−1^	[102]
	S-agCDs@PDA-MNPs-Ag NCs and a single-layer graphene substrate	SERS	NoV *	1 fg mL^−1^–10 ng mL^−1^	0.1 fg mL^−1^ (in PBS)	[112]
					0.95 fg mL^−1^ (in 10% human serum)	
				10–10^6^ RNA copies mL^−1^ (clinical NoV detection)	10 RNA copies mL^−1^	
		FL		10 fg mL^−1^–10 ng mL^−1^	5.8 fg mL^−1^ (in PBS)	
					6.5 fg mL^−1^ (in 10% human serum)	
				10^2^–10^6^ RNA copies mL^−1^ (clinical NoV detection)	80 RNA copies mL^−1^	
	Au@Ag/4-ATP@PDA@Ab and PDA-modified glass ship	SERS	migration inhibitory factor on exosome	5.44 × 10^2^–2.72 × 10^4^ particles/mL	one exosome in a 2 μL (9 × 10^−19^ mol L^−1^)	[115]
			glypican-1	5.44 × 10^2^–2.72 × 10^4^ particles/mL	9 × 10^−19^ mol L^−1^	
			epidermal growth factor receptor	5.44 × 10^2^–2.72 × 10^4^ particles/mL	9 × 10^−19^ mol L^−1^	
			CD63	2.72 × 10^3^–2.72 × 10^4^ particles/mL	4.5 × 10^−18^ mol L^−1^	
			EpCAM	5.44 × 10^2^–2.72 × 10^4^ particles/mL	9 × 10^−19^ mol L^−1^	
	Capillary glass@Au NSs@MIP	SERS	trypsin enzyme	0.01–1000 μg L^−1^	4.1 × 10^−3^ μg L^−1^	[117]
			pepsin	1 × 10^−3^–1000 μg L^−1^	0.6 × 10^−3^ μg L^−1^	
			BSA		0.4 × 10^−3^ μg L^−1^	
			hemoglobin		0.4 × 10^−3^ μg L^−1^	
	MPDA@Au-SAM and PVDF	SERS	miRNA-21	1 pM–10 μM	308.5 fM	[158]
	MPA@MB-P2 And AgNRs array electrode	SERS	miRNA-106a	100 fM–100 nM	67.44 fM	[159]
		EC			433.34 fM	
	Au-PS-PDA-Si chip	SERS	cTn Ⅰ	0.01–100 ng mL^−1^	3.16 pg mL^−1^	[163]
			creatine kinase isoenzyme MB		4.27 pg mL^−1^	
	Au@Ag/4-MPY^*^@BSA@PDA@Ab and PDA-modified glass chip	SERS	albumin	10–300 mg/L	0.2mg/L	[164]
	Au@MPB PDA-MIPs glass slide	SERS	acid phosphatase	18.2–1.82 × 10^6^ pM (1 ng mL^−1^–100 μg mL^−1^)	1.82 pM (0.1 ng mL^−1^)	[165]
			horseradish peroxidase	1 ng mL^−1^–100 μg mL^−1^	-	
			transferrin	0.1 ng mL^−1^–10 μg mL^−1^	-	
**Microfluidic devices**	Au NRs and MiChip *	SERS	prostate-specific antigen	0.1–100 ng mL^−1^	10 pg mL^−1^	[113]
			CEA			
			α-fetoprotein			
			*Escherichia coli O157:H7*	10^0^–10^4^ CFU μL^−1^	-	
			*Staphylococcus aureus*			
	Au NPs@NTP@Ag and MiChip		prostate-specific antigen	0–1 pg mL^−1^	0.2 pg mL^−1^	
**Lateral Flow devices**	Fe_3_O_4_@PDA@AuNPs	colorimetry	HCG *	0–500 mIU mL^−1^	10 mIU mL^−1^	[166]
		magnetic signal		0–500 mIU mL^−1^	1.2 mIU mL^−1^	
		SERS		0–50 mIU mL^−1^	0.2 mIU mL^−1^	
	PDA@Ag-NPs	SERS	SCCA *	10 pg mL^−1^–10 μg mL^−1^	7.156 pg mL^−1^ (in PBS)	[167]
					8.093 pg mL^−1^ (in human serum)	
			cancer antigen 125		7.182 pg mL^−1^ (in PBS)	
					7.370 pg mL^−1^ (in human serum)	

* ACE2: angiotensin-converting enzyme 2; cTn Ⅰ: cardiac troponin Ⅰ; PDR: PDA resin microspheres; EB: ethynylbenzene; GCMS: Au coated microarray substrate; 4-ATP: 4-aminothiophenol; MPB: 4-mercaptophenylboronic acid; CEA: carcinoembryonic antigen; NoV: norovirus; 4-MPY: 4-Mercaptopyridine; MiChip: microfluidic chip; HCG: human chorionic gonadotropin; SCCA: squamous cell carcinoma antigen.

## Data Availability

Data sharing not applicable.
